# Sustainable Development Goals as a Framework for Teaching and Learning about Health Equity in European Health and Social Care Study Programmes: A Modified Delphi Approach

**DOI:** 10.1007/s10916-025-02328-3

**Published:** 2025-12-22

**Authors:** Isabel Antón-Solanas, Fernando Urcola-Pardo, Ana B. Subirón-Valera, Davide Ziveri, Camilla Wikström-Grotell, Alessandra Aresu, Joost van Wijchen, Djenana Jalovcic, Cia Törnblom, Anu Nyberg, Beatriz Rodríguez-Roca, Maria Nordheim Alme

**Affiliations:** 1 Department of Physiatry and Nursing, Faculty of Health Sciences, University of Zaragoza, Zaragoza, Spain; 2 Research Group SAPIENF (B53_23R), University of Zaragoza, Zaragoza, Spain; 3 Handicap International - Humanity & Inclusion, Brussels, Belgium; 4 Department of Master Education and Research, Arcada UAS, Helsinki, Finland; 5 Department of Health and Welfare, Mälardalen University, Västerås, Sweden; 6 School of Allied Health, HAN University of Applied Sciences, Nijmegen, The Netherlands; 7 Department of Health and Functioning, Western Norway University of Applied Sciences, Bergen, Norway

**Keywords:** Health equity, Sustainable development goals, Delphi, Consensus, Health profession, Education

## Abstract

A health equity movement is underway, in which broad sectors of society must work together to create solutions to the complex interwoven problems that undermine equal opportunities for good health and well-being. Yet, addressing health inequity is a complex and challenging problem. Health inequity manifests through complex disparities that overload healthcare services and penetrate (all) other sectors of society. The aim of this study is to reach consensus on health equity related topics to be included in European health and social care study programmes by using the Sustainable Development Goals (SDGs). To identify such topics, a Delphi method was designed and performed in an expert panel comprising nine academics, clinicians, and members of a non-governmental organization. Using the Sustainable Development Goals as a framework, three rounds of surveys were conducted. The response rate was 100% across all rounds. In the first round, participants selected relevant SDG targets and indicators; 183 indicators were shortlisted. In the second round, participants rated the relevance of each indicator, leading to the endorsement of 142 indicators. In the third round, 162 out of 247 total indicators were endorsed. None of the Sustainable Development Goals were considered irrelevant to health and social care study programmes. We argue that to address health inequities effectively, health and social care professionals should liaise with a wide range of stakeholders in non-health sectors to design appropriate strategies to improve health and well-being. This implies that health and social care curricula should integrate competencies and capabilities that allow future professionals to work outside their traditional spheres of practice, communicating health information to a broad range of audiences, advocating and translating data for intersectoral action, and negotiating strategies and approaches to attain health equity in collaboration with stakeholders from different social sectors.

**Graphical Abstract:**

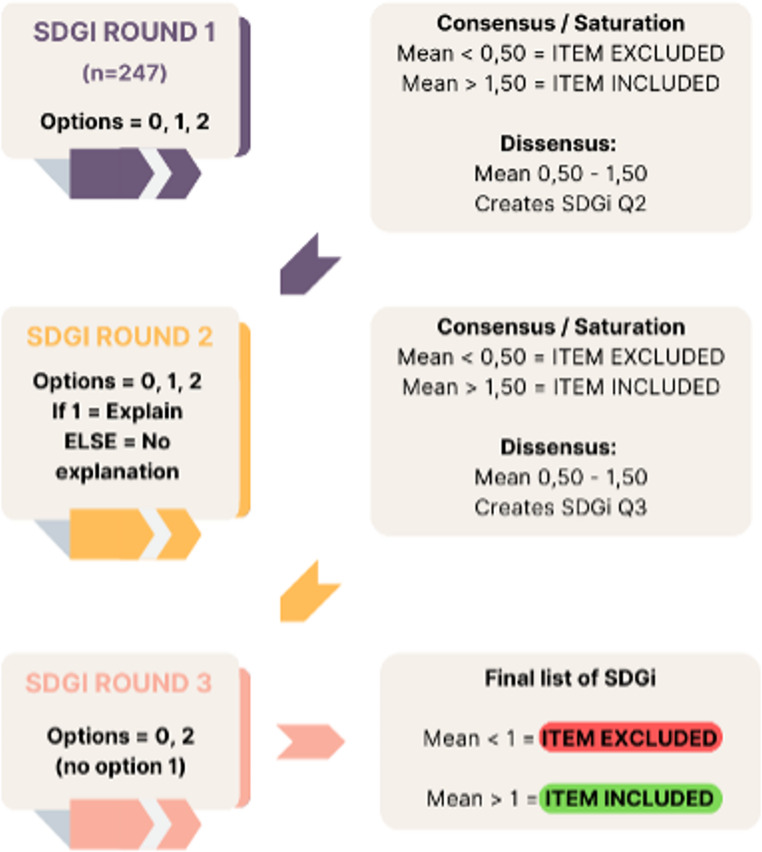

**Supplementary Information:**

The online version contains supplementary material available at 10.1007/s10916-025-02328-3.

## Introduction

Health equity is essential for creating a sustainable future and society. Based on moral values, the concept of equity points to a commitment to provide safe, meaningful, and appropriate care for all. Important for the preservation of sustainable societies is the accessibility of health services and the availability of equitable health care for all. Although European health systems are evolving in this direction for many people, there are still gaps that contribute to increasing health inequity in our societies.

Health equity is as complex as the very fabric of our society. A myriad of definitions of health equity have been proposed in the past decades. For instance, Aday et al. [1] viewed health equity from a resource allocation lens: “health care is equitable when resource allocation and access are determined by health needs”. From an outcome-oriented angle, Whitehead [2] suggested that “equity in health means that all persons have fair opportunities to attain their full health potential, to the extent possible”. With a view to measure and operationalize health equity, Braveman and Gruskin [3] argued that “equity in health is the absence of systematic disparities in health (or in the major social determinants of health) between groups with different levels of underlying social advantage/disadvantage, that is, wealth, power, or prestige. [Thus], assessing health equity requires comparing health and its social determinants between more and less advantaged social groups”. From a similar standpoint, but adding a layer of complexity, the World Health Organization (WHO) [4] defines health equity as an endpoint in which every individual can attain their full potential for health and well-being, and specifies that “health and health equity are determined by the conditions in which people are born, grow, live, work, play and age, as well as biological determinants” which, in turn, are conditioned by structural determinants (political, legal and economic) and ruled by social norms and institutional processes that shape the distribution of power and resources [5].

In addition, there are some recurrent concepts frequently associated with health equity. For instance, health equity is about confronting injustices and eliminating health disparities [6]; it is about protecting human rights [4]; it is about addressing social determinants of health [7]; it is about fair and just opportunity for all to attain their highest possible level of health [8]. However, health equity is not just about these, more familiar elements; health equity is evolving towards other, more recent concepts, such as intersectoral action [9], sustainability [10] and planetary health [11]. Whilst progress has been made in terms of defining health equity and tackling health inequity, there is little consensus about what this and other similar concepts mean (for example, “health disparity”, “health inequality” and “fair health”), and the resulting lack of clarity is both of academic and practical concern. How one defines these concepts can have important policy implications with a real impact on the population. For instance, it can determine which indicators are used to monitor people’s health and wellbeing, and which initiatives will receive support and resources to address these issues [12].

Endorsed by all United Nations (UN) member states in 2015, the 17 Sustainable Development Goals (SDGs) are at the heart of the 2030 Agenda for Sustainable Development [13], a global action plan that seeks to promote peace, prosperity, and sustainability for people and the planet, now and into the future. In the European context, the SDG framework has been adopted as a core reference for advancing equitable and sustainable development, aligned with long-standing regional commitments to human rights and social cohesion. The European Union has embedded the SDGs into major policy agendas, such as the European Green Deal and the European Pillar of Social Rights, to foster integrated action and position Europe as a leader in sustainable transformation. At the same time, persistent socio-economic disparities across member states highlight the need for continued efforts to ensure that SDG implementation strengthens cohesion within the region while contributing to global progress. The Agenda sets out 17 goals, 169 targets, and 247 indicators as a blueprint to address the world’s most pressing challenges. These goals are an urgent call for action to end poverty and other deprivations, improve health and education, spur economic growth and tackle climate change. With the use of 169 targets and 247 measurable indicators, the SDGs aim to give measures on how far we are on a global and national level in reaching the goals for fair and sustainable health and living. Further, the SDGs have a strong focus on inclusion, social justice and leaving no one behind [14]. Health equity resonates with these principles and provides a cross-cutting theme within this framework that can help policy makers develop coherent action across the sectoral goals and target areas [15, 16]. Yet, while the 17 SDGs present an inspiring vision of how the world could be, implementation is challenging. Specifically, action to attain the SDGs needs to cut across traditional sectors and consider how they impact on each other. In other words, social sectors must explore interconnectedness and maximize synergies by recognizing how policies in one area (such as education, housing, or transportation) can have indirect or direct effects on health outcomes. This requires intersectoral collaboration and the design of joint strategies that produce co-benefits across multiple domains of well-being and sustainable development.

While the approach presented here offers valuable insights for advancing SDG implementation and health equity, its transferability to other regions warrants careful consideration. Differences in governance structures, political priorities, socioeconomic conditions, and data availability may influence the feasibility and effectiveness of applying these strategies elsewhere. Rather than assuming direct replication, regions should adapt the framework to their institutional contexts, stakeholder landscapes, and existing policy tools. This may involve tailoring coordination mechanisms, aligning with local development agendas, and strengthening capacity for intersectoral collaboration and monitoring. By contextualizing and iteratively refining the process, other regions can build on the lessons generated here while ensuring relevance and sustainability within their own sociopolitical and institutional realities.

Universities are responsible for preparing future health and social care professionals, including those in Nursing, Medicine, Public Health, Social Work, Occupational Therapy, and other allied health disciplines. Accordingly, they must develop effective strategies to integrate concepts related to health equity into their curricula.

Traditionally, health and social care programmes emphasise domains closely aligned with clinical care and public health. For example, students are commonly exposed to topics linked to good health and well-being (SDG 3), gender equality (SDG 5), and clean water and sanitation (SDG 6). However, they are rarely introduced to SDGs less directly connected to clinical practice, such as sustainable cities and communities (SDG 11) or life on land (SDG 15). Although it is unrealistic for these programmes to cover all 17 SDGs in depth, exposure to a broader range of goals is essential, as many contribute indirectly to health equity and the social determinants of health. While the SDGs constitute a global framework, this study focuses on the European context. European Union policy has explicitly integrated the SDGs into health and social equity strategies, and health professional education in Europe is closely shaped by regional regulations, governance structures, and accreditation frameworks. As such, Europe provides a relevant context to explore how SDG-related content can be embedded in health and social care training. To address this need, we conducted a consultation process to identify which SDGs, targets, and indicators are most relevant for health and social care and should therefore be incorporated into academic curricula. The aim of this study was to examine the relevance of the SDGs to health equity and to identify key themes that should guide teaching and learning on health equity in health and social care programmes.

## Methodology

The present work has been driven by a panel of nine health equity experts in higher education (HE) from 5 European countries, namely Belgium, Finland, Norway, Spain and the Netherlands. The panel was composed of nine health and social care professionals working at four European universities and one non-governmental organization (NGO), all of them members of the EU-funded project health Equity through Education for a Sustainable Society (HEQED) (project reference: 2021-1-NO01-KA220-HED-000035777). All the experts had at least two years of experience in teaching and research on health equity and health equity-related concepts, and all had previously worked on research projects in these areas. Explicit inclusion criteria required: (i) a minimum of two years of academic experience teaching and conducting research on health equity or related concepts, and (ii) demonstrated participation in health equity research projects. Membership in relevant professional societies was considered supportive but was not used as a standalone inclusion criterion. The members of the panel’s sociodemographic characteristics are presented in Table 1.

**Table 1:** Sociodemographic characteristics of the members of the experts’ panel

Variable	*N* Mean	% SD
Age	50,4	7,2
Gender		
Female	6	66,7
Male	3	33,3
Professional affiliation		
HE	7	77,8
NGO	2	22,2
Professional background		
Nursing	2	22,2
Midwifery	2	22,2
Physiotherapy	1	11,1
Political science	1	11,1
Engineering	1	11,1
Environmental science	1	11,1
Neuroscience	1	11,1

Consensus procedures were agreed upon between the experts through e-mail correspondence and virtual meetings. In the first instance, an agreement was reached to select an existing, widely supported framework to identify indicators of health equity that is suitable to health and social care programmes; the SDG framework was selected by consensus.

**Fig. 1:**
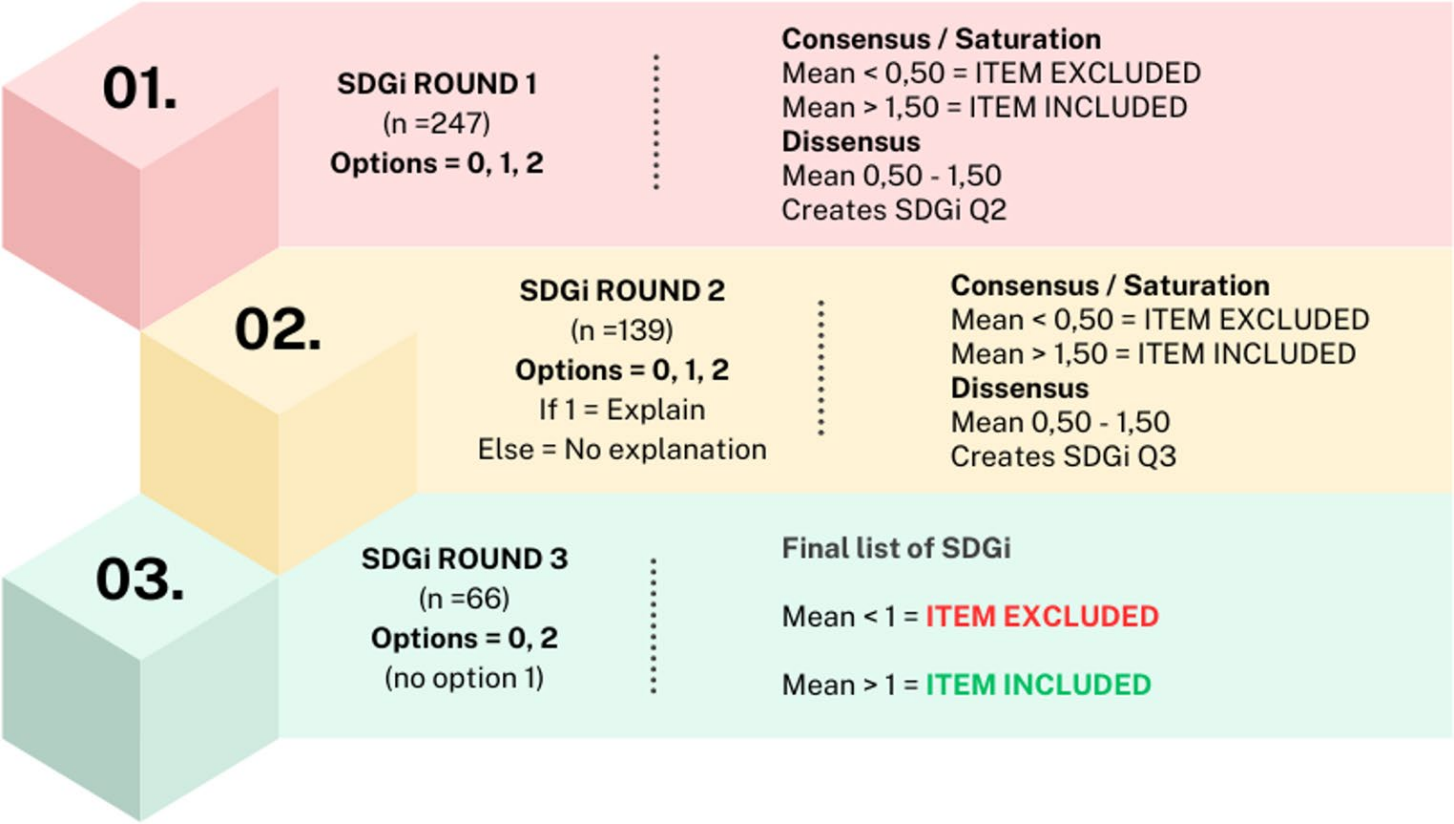
Sustainable Development Goals indicators (SDGi) analysis. Three survey rounds were performed rating the indicators as rejected (0), uncertain (1) or endorsed (2). From each round indicators were excluded (mean value< 0,50) or included (mean value >1,50. Indicators between 0,5 and 1,5 were defined as uncertain. These uncertain indicators were included in the next round

We conducted a survey using a structured, modified e-Delphi design [17] based on the 17 SDG and their respective 247 indicators defined by the UN on 16th July 2017 [18]. Three survey rounds were conducted to reach consensus between January and June 2022 (see Fig. 1). We used a modified (structured) e-Delphi method rather than a traditional Delphi approach because our process began with a predefined and comprehensive list of items (the 247 SDG indicators), rather than generating items inductively through open-ended questions in the first round. This allowed us to focus expert input on relevance and prioritization from the outset. All rounds were conducted online via surveys and virtual meetings, which is consistent with the characteristics of an e-Delphi methodology, and there were no losses.

A three-round Delphi process was conducted to identify SDG indicators considered relevant for measuring health inequities in Europe and suitable for integration into health and social care curricula. In the first round, experts were asked to assess the applicability and adequacy of each of the 247 SDG indicators to measure health inequities in the European context, as well as their potential integrability into European health and social care study programmes. Each indicator was rated using a three-point scale (0 = rejected, 1 = uncertain, 2 = endorsed). Indicators with a mean score > 1.50 were retained, those scoring < 0.50 were excluded, and those with scores between 0.50 and 1.50 were carried forward to a second round for reconsideration.

In the second round, the same rating criteria were applied. To support deeper consensus building, experts were additionally asked to briefly justify ratings of 1 (uncertain). As before, indicators with mean scores > 1.50 were retained, scores < 0.50 resulted in exclusion, and those with mean scores between 0.50 and 1.50 proceeded to a third round.

The comments from round two were synthesised and fed back anonymously to the panel in the third round alongside the relevant indicators. In this final round, experts were required to take a definitive stance and rate each remaining indicator as either 0 (rejected) or 2 (endorsed). Indicators were accepted if they achieved a mean score > 1. All ratings were completed independently and anonymously. Any lack of agreement was addressed through structured feedback and iterative re-rating rather than direct interaction, in line with established Delphi methodology. Stability across rounds was examined by assessing consistency in scoring ranges.

**Fig. 2:**
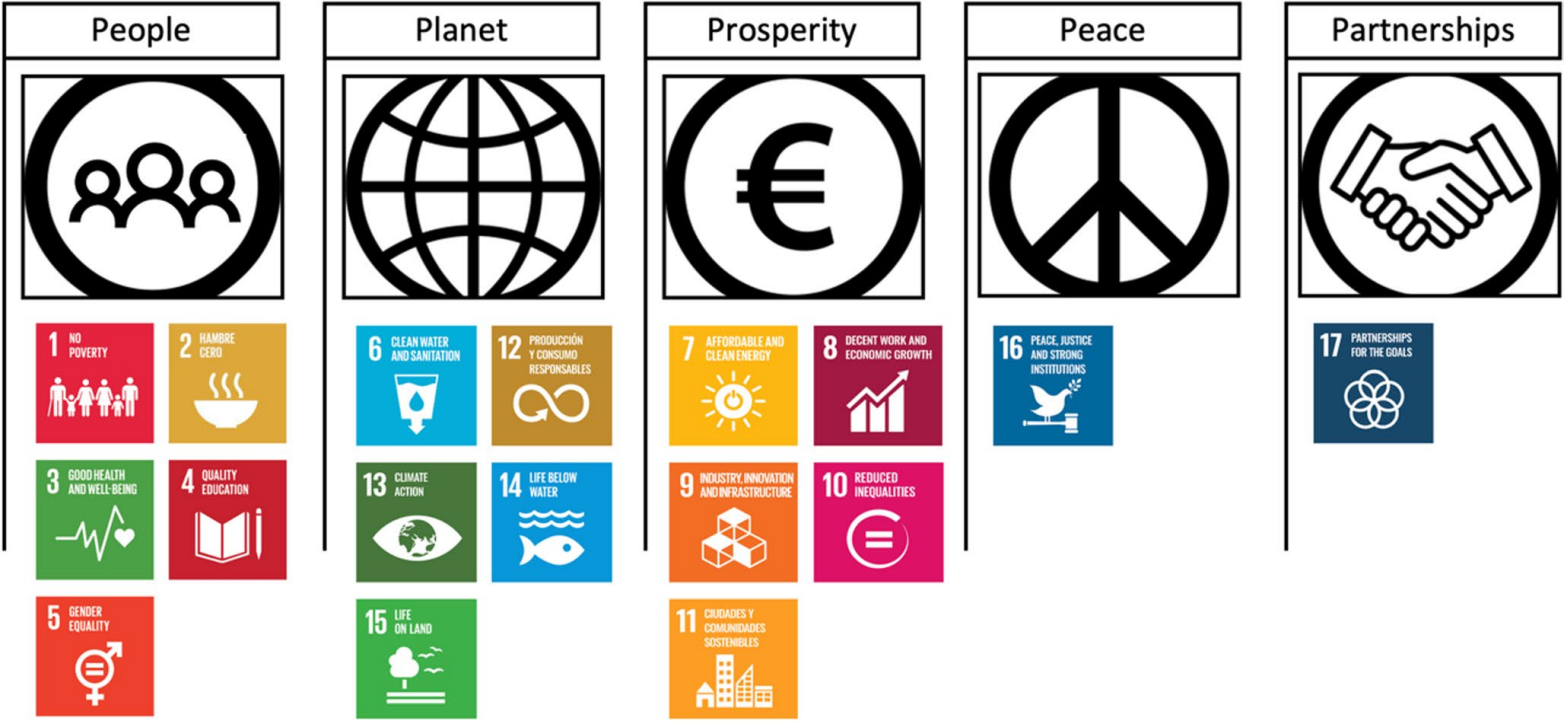
Classification of the SDG into the 5 pillars model. Source: modified from SDG Services (n.d.) [20]

Following the consensus process, the endorsed indicators were mapped onto the United Nations’ five-pillar SDG structure (people, planet, prosperity, peace, and partnership) to illustrate their alignment with global sustainable development priorities without implying normative interpretation (Department of Economic and Social Affairs & United Nations, 2015) [19], and depicted in Fig. 2.

This phase was guided by internationally recognised principles for effective international cooperation, including the Paris Principles [21], ensuring transparency, mutual accountability, and alignment with global governance standards. Detailed documentation of the indicator selection process is provided in Supplementary Files 1–4.

## Results

The initial survey included 247 indicators. After the first consultation round, 139 indicators remained. Following the second round, 66 indicators progressed to the final round and were ultimately endorsed (Table 2; see Supplementary File 1 for a complete list of goals, targets, and indicators across rounds).

**Table 2:** Indicators included, excluded, and re-rated after each survey round

Round	Indicators included	Indicators excluded	Indicators re-rated	Total evaluated indicators
1	48	60	139	247
2	64	9	66	139
3	50	16	-	66
Total	**162**	**85**		

Note: In Round 3, no indicators were re-rated as consensus was reached on all remaining items.

A total of 162 indicators were selected by the experts as adequate and applicable to build competence on health equity in the European context, and thus as relevant to health and social care curricula. All the indicators from Goal 4 were endorsed. In addition to Goal 4, Goals 1, 3 and 5 were widely supported by our panel. Goals 9, 13, 14, 15 and 17 received less support, with more than 50% of the indicators being excluded, respectively. Regardless of the percentage of indicators endorsed and rejected, not one single goal was perceived by the experts as being unrelated or irrelevant to health and social care professions (Table 3) (see Supplementary files 2–4 for a list of the indicators included, excluded, and re-rated after each round).

**Table 3:** Targets and indicators included for each goal based on the percentage of indicators included after phase 3

Goal	Targets	Targets endorsed	% of targets endorsed	Indicators	Indicators endorsed	% of indicators endorsed
4 Quality Education	10	10	100,00	12	12	100,00
3 Good Health and Well-being	13	13	100,00	28	27	96,43
5 Gender Equality	9	9	100,00	14	13	92,86
1 No Poverty	7	7	100,00	13	11	84,62
11 Sustainable Cities and Communities	10	8	80,00	14	11	78,57
16 Peace, Justice and Strong Institutions	12	9	75,00	24	18	75,00
6 Clean Water and Sanitation	8	7	87,50	11	8	72,73
10 Reduced Inequality	10	7	70,00	14	10	71,43
7 Affordable and Clean Energy	5	3	60,00	6	4	66,67
8 Decent Work and Economic Growth	12	8	66,67	16	10	62,50
12 Responsible Consumption and Production	11	6	54,55	13	7	53,85
2 Zero Hunger	8	4	50,00	14	7	50,00
15 Life on Land	12	5	41,67	14	6	42,86
9 Industry, Innovation and Infrastructure	8	5	62,50	12	5	41,67
13 Climate Action	5	3	60,00	8	3	37,50
17 Partnerships for the Goals	19	7	36,84	24	7	29,17
14 Life Below Water	10	1	10,00	10	1	10,00
TOTAL	**169**	**112**	**66**,**27**	**247**	**162**	**64**,**78**

To facilitate interpretation, the results are presented according to the UN five-pillar SDG structure. Overall, experts endorsed most indicators within the *People*, *Prosperity*, and *Peace* pillars, reflecting their perceived relevance to health equity and to health and social care education.

Regarding the goals classified under the People pillar, our experts gave it a high priority. They included every indicator for Goal 4 – Quality Education, and all but one of the indicators for Goal 3 – Good Health and Well-being and for Goal 5 – Gender Equality. Specifically, indicators 3.9.3 – Mortality rate attributed to unintentional poisoning and 5.a.1 – (*a*) Proportion of total agricultural population with ownership or secure rights over agricultural land, by sex; and (*b*) share of women among owners or rights-bearers of agricultural land, by type of tenure, were excluded by our expert panel. Only two indicators were excluded from Goal 1 – No Poverty. The topics covered by these two indicators included the total adult population with secure tenure rights to land, and the proportion of government spending on essential services and pro-poor public social spending. Finally, 50% of targets and indicators from Goal 2 – Zero Hunger were excluded after the third round. They were related to agricultural funding, production, export, and sustainability, as well as food price anomalies, plant and animal genetic resources for food and agriculture.

Regarding the SDG sustaining the Planet pillar, a large majority of the targets and indicators included in Goal 6 – Clean Water and Sanitation, were perceived as being relevant to health and social care study programmes. The indicators excluded were related to changes in water-related ecosystems, efficiency and transboundary basin areas. Only one indicator was seen as being relevant from Goal 14 – Life Below Water, that is 14.2.1 Number of countries using ecosystem-based approaches to managing marine areas. Approximately 40% of indicators from Goals 13 and Goal 15, and just over 50% of indicators from Goal 12 were endorsed by the expert panel. Topics pertaining to Goal 12 – Responsible production and Consumption included food loss and food waste, sustainable production and consumption, hazardous waste, recycling, global citizenship education for sustainable development and renewable energy-generating capacity; topics pertaining to Goal 13 – Climate Action included disaster risk reduction strategies, adaptation plans and communications on climate change and greenhouse gas emissions; finally, topics pertaining to Goal 15 – Life on Land included proportion of forest area and degraded land, sustainable forest management, and protected areas of terrestrial, freshwater and mountain biodiversity.

Most of the indicators pertaining to the Goals supporting the Prosperity pillar were endorsed by the expert panel. The topics addressed by the indicators included in Goal 7 – Affordable and Clean Energy included access to electricity, clean fuels and technology, renewable energy consumption and clean energy research. The topics endorsed from Goal 8 – Decent Work and Economic Growth included annual growth rate, level of national compliance with labour rights, rate of unemployment by sex, age, occupation and persons with disabilities, occupational injuries by sex and migrant status, proportion of informal employment by sector and sex, and number of children engaged in child labour. Under Goal 9 – Industry, Innovation and Infrastructure, the experts included the following topics: rural population living within 2 km of an all-season road, support to infrastructure, CO2 emissions and proportion of population covered by a mobile network and technology. Topics endorsed from Goal 10 – Reduced Inequality comprised growth rates of household expenditure or income per capita, proportion of people living below 50% of median income by sex, age and persons with disabilities, labour share of gross domestic product (GDP), redistributive impact of fiscal policy, migration policies that facilitate orderly, safe, regular and responsible migration, proportion of population personally feeling discriminated against or harassed and mobility of people and proportion of the population who are refugees. Finally, under Goal 11 - Sustainable Cities and Communities the following topics were endorsed: proportion of urban population living in slums, informal settlements or inadequate housing; access to public transport by sex, age and persons with disabilities; proportion of cities with, damage to infrastructure and disruption of basic services, and number of people dead or missing, attributed to disasters; average share of the built-up area of cities that is open space for public-use for all.

The Peace pillar is supported by Goal 16 – Peace, Justice, and Strong Institutions. Our experts endorsed 75% of the indicators included in this goal. Topics seen as relevant to health and social care study programmes included existence of independent national human rights institutions in compliance with the Paris Principles; proportion of population subjected to physical, psychological, sexual violence, discrimination and harassment, human trafficking and intentional homicide; number of conflict-related deaths by sex, age and cause; number of verified cases of killing, kidnapping, enforced disappearance, arbitrary detention and torture of journalists, associated media personnel, trade unionists and human rights advocates; number of unsentenced detainees; proportion of victims of violence who reported their case to the authorities; proportion of population satisfied with their last experience of public services; proportion of population that feel safe walking alone around the area they live; proportion of population who believe decision-making is inclusive and responsive, by sex, age, disability and population group; proportion of children under 5 years of age whose births have been registered; government expenditure by sector; and proportions of positions in national and local institutions, including (a) the legislatures; (b) the public service; and (c) the judiciary, compared to national distributions, by sex, age, persons with disabilities and population groups.

Finally, the Partnerships pillar is sustained by Goal 17 – Partnerships for the Goals. Only 30% of the indicators pertaining to Goal 17 were endorsed by the expert panel. Specifically, topics seen as relevant to health and social care study programmes included total government revenue; fixed Internet broadband subscriptions and proportion of persons using the Internet; mechanisms to enhance policy coherence and sustainable development; capacity for SDG monitoring; progress made in multi-stakeholder development effectiveness monitoring frameworks that support the achievement of the SDG; and proportion of countries that have conducted at least one population and housing census, have achieved 100 per cent and 80% birth and death registration, respectively.

## Discussion

Addressing health inequity is a complex and challenging problem. Health inequity manifests through complex disparities which overload healthcare services and penetrate (all) other sectors of society. Through a consensus process, this paper explores the SDGs as a model to address health equity in health and social work study programmes.

According to the WHO [22], it is the role and responsibility of health (and social) care to maintain good health and well-being. Therefore, the indicators pertaining to goals classified under the People pillar were selected as being relevant to health and social care professions. Whilst they are important, it is likely that some of the topics excluded by the panel were not perceived as being problematic in the European context; this is probably the case of indicators excluded from Goal 2 – Zero Hunger, which related mainly to agriculture. We wonder, however, whether this will change in the coming years due to the impact of climate change on crops and food production [23]. Also, some topics may have been perceived by the experts as being too far removed from health and social care professions’ area of influence; this may be the case of topics such as tenure rights to land and government spending on essential services. Yet, as stressed in the Adelaide Statement II on “Health in All Policies” [23], any political, economic, social and ecological decision has the capacity to affect health and well-being. Policies in virtually every sector of government can potentially increase or reduce health equity. Therefore, participation of the health sector in policy and decision-making is essential to successfully and effectively implement the Health in All Policies mandate [23].

According to The Lancet Public Health [24], planetary health and human health are inextricably connected. Whilst the planetary wellbeing is undoubtedly intimately linked to people’s health [25], only one indicator was seen as being relevant from Goal 14 – Life Below Water. Instead, aspects relating to clean water and sanitation, sustainable food production and consumption, waste production and recycling, sustainable forest management, protection of terrestrial, freshwater and mountain biodiversity, climate change, and global citizenship education and education for sustainable development, were perceived as being relevant to health and social care study programmes. Health professionals are increasingly committed to improving planetary health as a way to promote health and well-being. Therefore, we argue that planetary health should be a natural part of higher education for health and social care professionals [26].

According to Bauer [27], poor health is a burden on the economy at all levels. Our experts coincided with this assessment and endorsed a significant proportion of topics relating to prosperity. According to Frakt [28], “healthier” economies are associated with longer and healthier lives. However, the relationship between economic expansion and health is not always positive. There is a growing body of evidence that suggests that economic expansion is unhealthy [29] whilst, conversely, recessions can have a positive impact on some individuals’ health [30]. One theory suggests that, in time of economic growth, industries produce more air pollution and waste products, which exacerbates respiratory and other chronic conditions, and increases mortality [28]. Unfortunately, whilst sustainable economies have sometimes been identified with the ability to harness the earth, we should re-evaluate how we conduct business. When the natural world is utilised for human consumption, order in the ecological world becomes disturbed and this jeopardises public health [31]. One way to address this problem is adopting economic circular practices [32] and working towards sustainable green economic development [33]. In addition, it is important to consider that not everyone’s health improves or worsens equally in relation to economic changes. For example, a previous study [34] found an increase in mortality in the elderly, particularly older women living in nursing homes, during favourable economic conditions. The authors hypothesised that this was related to fluctuations in the number and professional skills of the nurses who these facilities were able to hire and retain. Other authors [35] have suggested that work can also play a key role in creating health inequity, for example, through exposure to occupational hazards and psychosocial risks. Other causes of morbidity and mortality in relation to work include exclusion from the labour market and worklessness. Some of the topics endorsed by our experts touched on some of these issues, including clean and renewable energy, labour rights and employment, housing and infrastructure, among others. Thus, we argue that health and social care professionals should have the opportunity to learn about the relationship between economy (or prosperity) and how it impacts on health, both in favourable and adverse conditions.

Unsurprisingly, the indicators included in Goal 16 – Peace and Justice Strong Institutions were strongly endorsed by the experts. Topics seen as relevant to health and social care curricula included human rights, types and prevalence of violence, effectiveness of public services, and government expenditure by sector. The relationship between health equity and peace is clear: there can be no health (or health equity) without peace [36]. In the European context, this connection has become increasingly visible. Ongoing conflicts at Europe’s borders, political instability in neighbouring regions, and rising social and political tensions within Europe have contributed to a growing number of forcibly displaced people seeking safety. Migrants and refugees face disproportionate barriers to healthcare access, higher rates of poor mental health, increased exposure to violence and exploitation, and elevated risks of morbidity and premature mortality. Similarly, gender-based violence, discrimination, and systemic inequities continue to affect women across Europe, limiting their opportunities to achieve optimal health and well-being. Without peace, safety, and effective institutions, these groups bear the greatest burden of suffering and avoidable harm. According to Wesley et al. [37], health professionals are morally obliged not only to recognise the inextricable link between peace and health equity, but also to actively contribute to preventing violence and fostering peace at all levels—from policy advocacy to clinical encounters. Violence and injustice occur not only on the global or national stage; they also manifest locally and within families, constraining individuals’ ability to live healthy and fulfilled lives. For these reasons, we argue that health and social care professionals must be equipped to understand and address the intersection between peace, human rights, and health equity as part of their professional formation.

In practical terms, achieving health equity in Europe means that structurally disadvantaged groups—particularly migrants, refugees and women—can access timely, affordable and culturally appropriate care and live in conditions that do not undermine health. When this is not achieved, preventable suffering increases and avoidable morbidity and mortality rise. Evidence shows higher risks of poor mental health, infectious disease, and maternal/perinatal complications among refugees and migrants—compounded by barriers to care and exposure to violence and exploitation [38–40]. For women, persistent gender inequalities and high levels of gender-based violence continue to drive health gaps across the life course [41, 42]. In the current European context—marked by geopolitical instability, large-scale displacement, and the cost-of-living crisis—addressing health equity is urgent to prevent widening disparities and protect population health [43, 44].

More surprising was the fact that only 30% of the indicators included in Goal 17 – Partnerships to Achieve the Goal were endorsed by the experts. These included aspects as crucial as revenue, access to technology, capacity for SDG monitoring and strategies for intersectoral and multistakeholder collaboration and monitoring. Some of the indicators excluded by the panel were designed to measure, for example, the “worldwide weighted tariff-average”, and “developing countries and least developed countries’ share of global exports”. Whilst it is obvious that achieving the SDGs, and thus furthering health equity, depends on international, intersectoral and multi-stakeholder collaboration [45], the indicators collected under Goal 17 are pitched at a macro level. Therefore, it is possible that they were not endorsed due to being perceived as not relevant to undergraduate and even postgraduate health and social care students.

In this study, none of the 17 SDGs were perceived by the experts as being inadequate, irrelevant, or unapplicable to European health and social work study programmes. A wide range of different indicators, pertaining to a variety of targets and goals, were identified as being useful to address health (in)equity in higher education. This finding sparked a process of reflection, which resulted in three key arguments: (1) health equity is not circumscribed to healthcare, but it is a shared responsibility between healthcare professionals and the wider society; (2) as experts in healthcare, health and social care professionals should expand their role and exceed their traditional areas of practice in order to work collaboratively with other sectors of society; and (3) achieving health equity is not an end in itself, but a complex process, in which healthcare professionals must become involved. This includes not only integrating health equity into one’s practice, but also liaising with a wide range of stakeholders and non-health sectors to design appropriate strategies to improve health and well-being. Yet, cross-sectoral collaboration to reduce health inequalities is not straightforward. We argue that health and social care study programmes should train students to become involved in resource management and decision making to promote health equity, to communicate health information to a broad range of audiences, to advocate and translate data for intersectoral action, and to negotiate strategies and approaches to attain health equity in collaboration with stakeholders from different social sectors.

## Conclusion

In this paper, the 17 SDG have been used as a framework to identify topics that ought to be integrated into health and social care study programmes in order to build competence in promoting health equity. Through a Delphi process, expert consensus was achieved on the topics that are relevant for health and social care students’ training. The indicators pertaining to the goals that support the People, Prosperity and Peace pillars were perceived as being strongly linked to health equity. Whilst fewer indicators pertaining to the goals sustaining the Planet and Partnerships pillars were endorsed by the members of the panel, none of the 17 SDG were perceived as being irrelevant or unrelated. Many of the topics endorsed by our panel have traditionally been part of health and social care study programmes; for example, the mortality rate attributed to cardiovascular disease, cancer, diabetes or chronic respiratory disease. However, others have rarely (or never) been integrated before, for example, the proportion of bodies of water with good ambient water quality. In view of our results, we argue that (1) health equity is not circumscribed to health care only, (2) health and social care professionals should expand their role and exceed their traditional areas of practice and (3) health equity is a continuous process that is heavily influenced by the political, societal, and environmental factors. We argue that health and social care professionals should learn to balance health considerations with the goals of other social sectors in order to maximize co-benefits. Yet, this requires specific knowledge, skills and competencies, which are not currently integrated into study programmes. We argue that higher education institutions should strengthen this focus in order to expand the role of health and social care professionals.

Despite the valuable insights gained, several limitations should be acknowledged. First, the expert panel consisted of nine members, which may limit the generalizability of the findings to broader contexts or different geographical regions. Additionally, while the Delphi process achieved consensus, the perspectives of other stakeholders, such as patients, policy makers, or educators, were not directly included and could provide complementary insights. Future research could explore the integration of these viewpoints to further refine and validate the identified topics.

Moreover, ongoing work is needed to translate these consensus-based topics into concrete curricular modules and teaching strategies. Evaluating the effectiveness of incorporating these competencies in health and social care education, and their subsequent impact on professional practice and health equity outcomes, remains an open and critical area for investigation. Finally, adapting curricula to rapidly evolving social, environmental, and political contexts will require continuous monitoring and flexibility.

## Supplementary Information

Below is the link to the electronic supplementary material.

Supplementary Material 1 (DOCX 39.5 KB)

Supplementary Material 2 (DOCX 36.7 KB)

Supplementary Material 3 (DOCX 26.5 KB)

Supplementary Material 4 (DOCX 19.6 KB)

## Data Availability

No datasets were generated or analysed during the current study.
