# Supplementary material for: Sustainable Development Goals as a Framework for Teaching and Learning about Health Equity in European Health and Social Care Study Programmes: A Modified Delphi Approach
Source: J Med Syst. 2025 Dec 22;49(1):187. doi: 10.1007/s10916-025-02328-3 (PMC12722463; doi:10.1007/s10916-025-02328-3)
Supplement: Supplementary file 1 — Supplementary Material 1 (DOCX 39.5 KB) [file 10916_2025_2328_MOESM1_ESM.docx]

**SUPPLEMENTARY FILE 1: INDICATORS ENDORSED**

**Supplementary file 1** presents all the indicators included after each round: Round 1 = blue; Round 2 = red; Round 3 = green by goal, from 1 to 17.

| ***Goals and targets (from the 2030 Agenda for Sustainable Development)*** | ***Indicators*** |
| --- | --- |
| **Goal 1. End poverty in all its forms everywhere** | |
| 1.1 By 2030, eradicate extreme poverty for all people everywhere, currently measured as people living on less than $1.25 a day | 1.1.1 Proportion of the population living below the international poverty line by sex, age, employment status and geographic location (urban/rural) |
| 1.2 By 2030, reduce at least by half the proportion of men, women and children of all ages living in poverty in all its dimensions according to national definitions | 1.2.1 Proportion of population living below the national poverty line, by sex and age |
|  | 1.2.2 Proportion of men, women and children of all ages living in poverty in all its dimensions according to national definitions |
| 1.3 Implement nationally appropriate social protection systems and measures for all, including floors, and by 2030 achieve substantial coverage of the poor and the vulnerable | 1.3.1 Proportion of population covered by social protection floors/systems, by sex, distinguishing children, unemployed persons, older persons, persons with disabilities, pregnant women, newborns, work-injury victims and the poor and the vulnerable |
| 1.4 By 2030, ensure that all men and women, in particular the poor and the vulnerable, have equal rights to economic resources, as well as access to basic services, ownership and control over land and other forms of property, inheritance, natural resources, appropriate new technology and financial services, including microfinance | 1.4.1 Proportion of population living in households with access to basic services |
|  | 1.4.2 Proportion of total adult population with secure tenure rights to land, (*a*) with legally recognized documentation, and (*b*) who perceive their rights to land as secure, by sex and type of tenure |
| 1.5 By 2030, build the resilience of the poor and those in vulnerable situations and reduce their exposure and vulnerability to climate-related extreme events and other economic, social and environmental shocks and disasters | 1.5.1 Number of deaths, missing persons and directly affected persons attributed to disasters per 100,000 population |
|  | 1.5.3 Number of countries that adopt and implement national disaster risk reduction strategies in line with the Sendai Framework for Disaster Risk Reduction 2015–2030 |
| 1.a Ensure significant mobilization of resources from a variety of sources, including through enhanced development cooperation, in order to provide adequate and predictable means for developing countries, in particular least developed countries, to implement programmes and policies to end poverty in all its dimensions | 1.a.1 Total official development assistance grants from all donors that focus on poverty reduction as a share of the recipient country’s gross national income |
|  | 1.a.2 Proportion of total government spending on essential services (education, health and social protection) |
| 1.b Create sound policy frameworks at the national, regional and international levels, based on pro-poor and gender-sensitive development strategies, to support accelerated investment in poverty eradication actions | 1.b.1 Pro-poor public social spending |

| ***Goals and targets (from the 2030 Agenda for Sustainable Development)*** | ***Indicators*** |
| --- | --- |
| **Goal 2. End hunger, achieve food security and improved nutrition and promote sustainable agriculture** | |
| 2.1 By 2030, end hunger and ensure access by all people, in particular the poor and people in vulnerable situations, including infants, to safe, nutritious and sufficient food all year round | 2.1.1 Prevalence of undernourishment |
|  | 2.1.2 Prevalence of moderate or severe food insecurity in the population, based on the Food Insecurity Experience Scale (FIES) |
| 2.2 By 2030, end all forms of malnutrition, including achieving, by 2025, the internationally agreed targets on stunting and wasting in children under 5 years of age, and address the nutritional needs of adolescent girls, pregnant and lactating women and older persons | 2.2.1 Prevalence of stunting (height for age <-2 standard deviation from the median of the World Health Organization (WHO) Child Growth Standards) among children under 5 years of age |
|  | 2.2.2 Prevalence of malnutrition (weight for height >+2 or <-2 standard deviation from the median of the WHO Child Growth Standards) among children under 5 years of age, by type (wasting and overweight) |
|  | 2.2.3 Prevalence of anaemia in women aged 15 to 49 years, by pregnancy status (percentage) |
| 2.4 By 2030, ensure sustainable food production systems and implement resilient agricultural practices that increase productivity and production, that help maintain ecosystems, that strengthen capacity for adaptation to climate change, extreme weather, drought, flooding and other disasters and that progressively improve land and soil quality | 2.4.1 Proportion of agricultural area under productive and sustainable agriculture |
| 2.c Adopt measures to ensure the proper functioning of food commodity markets and their derivatives and facilitate timely access to market information, including on food reserves, in order to help limit extreme food price volatility | 2.c.1 Indicator of food price anomalies |

| ***Goals and targets (from the 2030 Agenda for Sustainable Development)*** | ***Indicators*** |
| --- | --- |
| **Goal 3. Ensure healthy lives and promote well-being for all at all ages** | |
| 3.1 By 2030, reduce the global maternal mortality ratio to less than 70 per 100,000 live births | 3.1.1 Maternal mortality ratio |
|  | 3.1.2 Proportion of births attended by skilled health personnel |
| 3.2 By 2030, end preventable deaths of newborns and children under 5 years of age, with all countries aiming to reduce neonatal mortality to at least as low as 12 per 1,000 live births and under5 mortality to at least as low as 25 per 1,000 live births | 3.2.1 Under5 mortality rate |
|  | 3.2.2 Neonatal mortality rate |
| 3.3 By 2030, end the epidemics of AIDS, tuberculosis, malaria and neglected tropical diseases and combat hepatitis, water-borne diseases and other communicable diseases | 3.3.1 Number of new HIV infections per 1,000 uninfected population, by sex, age and key populations |
|  | 3.3.2 Tuberculosis incidence per 100,000 population |
|  | 3.3.3 Malaria incidence per 1,000 population |
|  | 3.3.4 Hepatitis B incidence per 100,000 population |
|  | 3.3.5 Number of people requiring interventions against neglected tropical diseases |
| 3.4 By 2030, reduce by one third premature mortality from non-communicable diseases through prevention and treatment and promote mental health and well-being | 3.4.1 Mortality rate attributed to cardiovascular disease, cancer, diabetes or chronic respiratory disease |
|  | 3.4.2 Suicide mortality rate |
| 3.5 Strengthen the prevention and treatment of substance abuse, including narcotic drug abuse and harmful use of alcohol | 3.5.1 Coverage of treatment interventions (pharmacological, psychosocial and rehabilitation and aftercare services) for substance use disorders |
|  | 3.5.2 Alcohol per capita consumption (aged 15 years and older) within a calendar year in litres of pure alcohol |
| 3.6 By 2020, halve the number of global deaths and injuries from road traffic accidents | 3.6.1 Death rate due to road traffic injuries |
| 3.7 By 2030, ensure universal access to sexual and reproductive health-care services, including for family planning, information and education, and the integration of reproductive health into national strategies and programmes | 3.7.1 Proportion of women of reproductive age (aged 15–49 years) who have their need for family planning satisfied with modern methods |
|  | 3.7.2 Adolescent birth rate (aged 10–14 years; aged 15–19 years) per 1,000 women in that age group |
| 3.8 Achieve universal health coverage, including financial risk protection, access to quality essential health-care services and access to safe, effective, quality and affordable essential medicines and vaccines for all | 3.8.1 Coverage of essential health services |
|  | 3.8.2 Proportion of population with large household expenditures on health as a share of total household expenditure or income |
| 3.9 By 2030, substantially reduce the number of deaths and illnesses from hazardous chemicals and air, water and soil pollution and contamination | 3.9.1 Mortality rate attributed to household and ambient air pollution |
|  | 3.9.2 Mortality rate attributed to unsafe water, unsafe sanitation and lack of hygiene (exposure to unsafe Water, Sanitation and Hygiene for All (WASH) services) |
| 3.a Strengthen the implementation of the World Health Organization Framework Convention on Tobacco Control in all countries, as appropriate | 3.a.1 Age-standardized prevalence of current tobacco use among persons aged 15 years and older |
| 3.b Support the research and development of vaccines and medicines for the communicable and noncommunicable diseases that primarily affect developing countries, provide access to affordable essential medicines and vaccines, in accordance with the Doha Declaration on the TRIPS Agreement and Public Health, which affirms the right of developing countries to use to the full the provisions in the Agreement on Trade-Related Aspects of Intellectual Property Rights regarding flexibilities to protect public health, and, in particular, provide access to medicines for all | 3.b.1 Proportion of the target population covered by all vaccines included in their national programme |
|  | 3.b.2 Total net official development assistance to medical research and basic health sectors |
|  | 3.b.3 Proportion of health facilities that have a core set of relevant essential medicines available and affordable on a sustainable basis |
| 3.c Substantially increase health financing and the recruitment, development, training and retention of the health workforce in developing countries, especially in least developed countries and small island developing States | 3.c.1 Health worker density and distribution |
| 3.d Strengthen the capacity of all countries, in particular developing countries, for early warning, risk reduction and management of national and global health risks | 3.d.1 International Health Regulations (IHR) capacity and health emergency preparedness |
|  | 3.d.2 Percentage of bloodstream infections due to selected antimicrobial-resistant organisms |

| ***Goals and targets (from the 2030 Agenda for Sustainable Development)*** | ***Indicators*** |
| --- | --- |
| **Goal 4. Ensure inclusive and equitable quality education and promote lifelong learning opportunities for all** | |
| 4.1 By 2030, ensure that all girls and boys complete free, equitable and quality primary and secondary education leading to relevant and effective learning outcomes | 4.1.1 Proportion of children and young people (*a*) in grades 2/3; (*b*) at the end of primary; and (*c*) at the end of lower secondary achieving at least a minimum proficiency level in (i) reading and (ii) mathematics, by sex |
|  | 4.1.2 Completion rate (primary education, lower secondary education, upper secondary education) |
| 4.2 By 2030, ensure that all girls and boys have access to quality early childhood development, care and preprimary education so that they are ready for primary education | 4.2.1 Proportion of children aged 24–59 months who are developmentally on track in health, learning and psychosocial well-being, by sex |
|  | 4.2.2 Participation rate in organized learning (one year before the official primary entry age), by sex |
| 4.3 By 2030, ensure equal access for all women and men to affordable and quality technical, vocational and tertiary education, including university | 4.3.1 Participation rate of youth and adults in formal and non-formal education and training in the previous 12 months, by sex |
| 4.4 By 2030, substantially increase the number of youth and adults who have relevant skills, including technical and vocational skills, for employment, decent jobs and entrepreneurship | 4.4.1 Proportion of youth and adults with information and communications technology (ICT) skills, by type of skill |
| 4.5 By 2030, eliminate gender disparities in education and ensure equal access to all levels of education and vocational training for the vulnerable, including persons with disabilities, indigenous peoples and children in vulnerable situations | 4.5.1 Parity indices (female/male, rural/urban, bottom/top wealth quintile and others such as disability status, indigenous peoples and conflict-affected, as data become available) for all education indicators on this list that can be disaggregated |
| 4.6 By 2030, ensure that all youth and a substantial proportion of adults, both men and women, achieve literacy and numeracy | 4.6.1 Proportion of population in a given age group achieving at least a fixed level of proficiency in functional (*a*) literacy and (*b*) numeracy skills, by sex |
| 4.7 By 2030, ensure that all learners acquire the knowledge and skills needed to promote sustainable development, including, among others, through education for sustainable development and sustainable lifestyles, human rights, gender equality, promotion of a culture of peace and non-violence, global citizenship and appreciation of cultural diversity and of culture’s contribution to sustainable development | 4.7.1 Extent to which (i) global citizenship education and (ii) education for sustainable development are mainstreamed in (*a*) national education policies; (*b*) curricula; (*c*) teacher education; and (*d*) student assessment |
| 4.a Build and upgrade education facilities that are child, disability and gender sensitive and provide safe, non-violent, inclusive and effective learning environments for all | 4.a.1 Proportion of schools offering basic services, by type of service |
| 4.b By 2020, substantially expand globally the number of scholarships available to developing countries, in particular least developed countries, small island developing States and African countries, for enrolment in higher education, including vocational training and information and communications technology, technical, engineering and scientific programmes, in developed countries and other developing countries | 4.b.1 Volume of official development assistance flows for scholarships by sector and type of study |
| 4.c By 2030, substantially increase the supply of qualified teachers, including through international cooperation for teacher training in developing countries, especially least developed countries and small island developing States | 4.c.1 Proportion of teachers with the minimum required qualifications, by education level |

| ***Goals and targets (from the 2030 Agenda for Sustainable Development)*** | ***Indicators*** |
| --- | --- |
| **Goal 5. Achieve gender equality and empower all women and girls** | |
| 5.1 End all forms of discrimination against all women and girls everywhere | 5.1.1 Whether or not legal frameworks are in place to promote, enforce and monitor equality and nondiscrimination on the basis of sex |
| 5.2 Eliminate all forms of violence against all women and girls in the public and private spheres, including trafficking and sexual and other types of exploitation | 5.2.1 Proportion of ever-partnered women and girls aged 15 years and older subjected to physical, sexual or psychological violence by a current or former intimate partner in the previous 12 months, by form of violence and by age |
|  | 5.2.2 Proportion of women and girls aged 15 years and older subjected to sexual violence by persons other than an intimate partner in the previous 12 months, by age and place of occurrence |
| 5.3 Eliminate all harmful practices, such as child, early and forced marriage and female genital mutilation | 5.3.1 Proportion of women aged 20–24 years who were married or in a union before age 15 and before age 18 |
|  | 5.3.2 Proportion of girls and women aged 15–49 years who have undergone female genital mutilation/cutting, by age |
| 5.4 Recognize and value unpaid care and domestic work through the provision of public services, infrastructure and social protection policies and the promotion of shared responsibility within the household and the family as nationally appropriate | 5.4.1 Proportion of time spent on unpaid domestic and care work, by sex, age and location |
| 5.5 Ensure women’s full and effective participation and equal opportunities for leadership at all levels of decision-making in political, economic and public life | 5.5.1 Proportion of seats held by women in (*a*) national parliaments and (*b*) local governments |
|  | 5.5.2 Proportion of women in managerial positions |
| 5.6 Ensure universal access to sexual and reproductive health and reproductive rights as agreed in accordance with the Programme of Action of the International Conference on Population and Development and the Beijing Platform for Action and the outcome documents of their review conferences | 5.6.1 Proportion of women aged 15–49 years who make their own informed decisions regarding sexual relations, contraceptive use and reproductive health care |
|  | 5.6.2 Number of countries with laws and regulations that guarantee full and equal access to women and men aged 15 years and older to sexual and reproductive health care, information and education |
| 5.a Undertake reforms to give women equal rights to economic resources, as well as access to ownership and control over land and other forms of property, financial services, inheritance and natural resources, in accordance with national laws | 5.a.2 Proportion of countries where the legal framework (including customary law) guarantees women’s equal rights to land ownership and/or control |
| 5.b Enhance the use of enabling technology, in particular information and communications technology, to promote the empowerment of women | 5.b.1 Proportion of individuals who own a mobile telephone, by sex |
| 5.c Adopt and strengthen sound policies and enforceable legislation for the promotion of gender equality and the empowerment of all women and girls at all levels | 5.c.1 Proportion of countries with systems to track and make public allocations for gender equality and women’s empowerment |

| ***Goals and targets (from the 2030 Agenda for Sustainable Development)*** | ***Indicators*** |
| --- | --- |
| **Goal 6. Ensure availability and sustainable management of water and sanitation for all** | |
| 6.1 By 2030, achieve universal and equitable access to safe and affordable drinking water for all | 6.1.1 Proportion of population using safely managed drinking water services |
| 6.2 By 2030, achieve access to adequate and equitable sanitation and hygiene for all and end open defecation, paying special attention to the needs of women and girls and those in vulnerable situations | 6.2.1 Proportion of population using (*a*) safely managed sanitation services and (*b*) a hand-washing facility with soap and water |
| 6.3 By 2030, improve water quality by reducing pollution, eliminating dumping and minimizing release of hazardous chemicals and materials, halving the proportion of untreated wastewater and substantially increasing recycling and safe reuse globally | 6.3.1 Proportion of domestic and industrial wastewater flows safely treated |
|  | 6.3.2 Proportion of bodies of water with good ambient water quality |
| 6.4 By 2030, substantially increase water-use efficiency across all sectors and ensure sustainable withdrawals and supply of freshwater to address water scarcity and substantially reduce the number of people suffering from water scarcity | 6.4.2 Level of water stress: freshwater withdrawal as a proportion of available freshwater resources |
| 6.5 By 2030, implement integrated water resources management at all levels, including through transboundary cooperation as appropriate | 6.5.1 Degree of integrated water resources management |
| 6.a By 2030, expand international cooperation and capacity-building support to developing countries in water- and sanitation-related activities and programmes, including water harvesting, desalination, water efficiency, wastewater treatment, recycling and reuse technologies | 6.a.1 Amount of water- and sanitation-related official development assistance that is part of a government-coordinated spending plan |
| 6.b Support and strengthen the participation of local communities in improving water and sanitation management | 6.b.1 Proportion of local administrative units with established and operational policies and procedures for participation of local communities in water and sanitation management |

| ***Goals and targets (from the 2030 Agenda for Sustainable Development)*** | ***Indicators*** |
| --- | --- |
| **Goal 7. Ensure access to affordable, reliable, sustainable and modern energy for all** | |
| 7.1 By 2030, ensure universal access to affordable, reliable and modern energy services | 7.1.1 Proportion of population with access to electricity |
|  | 7.1.2 Proportion of population with primary reliance on clean fuels and technology |
| 7.2 By 2030, increase substantially the share of renewable energy in the global energy mix | 7.2.1 Renewable energy share in the total final energy consumption |
| 7.a By 2030, enhance international cooperation to facilitate access to clean energy research and technology, including renewable energy, energy efficiency and advanced and cleaner fossil-fuel technology, and promote investment in energy infrastructure and clean energy technology | 7.a.1 International financial flows to developing countries in support of clean energy research and development and renewable energy production, including in hybrid systems |

| ***Goals and targets (from the 2030 Agenda for Sustainable Development)*** | ***Indicators*** |
| --- | --- |
| **Goal 8. Promote sustained, inclusive and sustainable economic growth, full and productive employment and decent work for all** | |
| 8.1 Sustain per capita economic growth in accordance with national circumstances and, in particular, at least 7 per cent gross domestic product growth per annum in the least developed countries | 8.1.1 Annual growth rate of real GDP per capita |
| 8.2 Achieve higher levels of economic productivity through diversification, technological upgrading and innovation, including through a focus on high-value added and labour-intensive sectors | 8.2.1 Annual growth rate of real GDP per employed person |
| 8.3 Promote development-oriented policies that support productive activities, decent job creation, entrepreneurship, creativity and innovation, and encourage the formalization and growth of micro-, small- and medium-sized enterprises, including through access to financial services | 8.3.1 Proportion of informal employment in total employment, by sector and sex |
| 8.5 By 2030, achieve full and productive employment and decent work for all women and men, including for young people and persons with disabilities, and equal pay for work of equal value | 8.5.1 Average hourly earnings of employees, by sex, age, occupation and persons with disabilities |
|  | 8.5.2 Unemployment rate, by sex, age and persons with disabilities |
| 8.6 By 2020, substantially reduce the proportion of youth not in employment, education or training | 8.6.1 Proportion of youth (aged 15–24 years) not in education, employment or training |
| 8.7 Take immediate and effective measures to eradicate forced labour, end modern slavery and human trafficking and secure the prohibition and elimination of the worst forms of child labour, including recruitment and use of child soldiers, and by 2025 end child labour in all its forms | 8.7.1 Proportion and number of children aged 5–17 years engaged in child labour, by sex and age |
| 8.8 Protect labour rights and promote safe and secure working environments for all workers, including migrant workers, in particular women migrants, and those in precarious employment | 8.8.1 Fatal and non-fatal occupational injuries per 100,000 workers, by sex and migrant status |
|  | 8.8.2 Level of national compliance with labour rights (freedom of association and collective bargaining) based on International Labour Organization (ILO) textual sources and national legislation, by sex and migrant status |
| 8.b By 2020, develop and operationalize a global strategy for youth employment and implement the Global Jobs Pact of the International Labour Organization | 8.b.1 Existence of a developed and operationalized national strategy for youth employment, as a distinct strategy or as part of a national employment strategy |

| ***Goals and targets (from the 2030 Agenda for Sustainable Development)*** | ***Indicators*** |
| --- | --- |
| **Goal 9. Build resilient infrastructure, promote inclusive and sustainable industrialization and foster innovation** | |
| 9.1 Develop quality, reliable, sustainable and resilient infrastructure, including regional and transborder infrastructure, to support economic development and human well-being, with a focus on affordable and equitable access for all | 9.1.1 Proportion of the rural population who live within 2 km of an all-season road |
| 9.4 By 2030, upgrade infrastructure and retrofit industries to make them sustainable, with increased resource-use efficiency and greater adoption of clean and environmentally sound technologies and industrial processes, with all countries taking action in accordance with their respective capabilities | 9.4.1 CO_2_ emission per unit of value added |
| 9.5 Enhance scientific research, upgrade the technological capabilities of industrial sectors in all countries, in particular developing countries, including, by 2030, encouraging innovation and substantially increasing the number of research and development workers per 1 million people and public and private research and development spending | 9.5.2 Researchers (in full-time equivalent) per million inhabitants |
| 9.a Facilitate sustainable and resilient infrastructure development in developing countries through enhanced financial, technological and technical support to African countries, least developed countries, landlocked developing countries and small island developing States | 9.a.1 Total official international support (official development assistance plus other official flows) to infrastructure |
| 9.c Significantly increase access to information and communications technology and strive to provide universal and affordable access to the Internet in least developed countries by 2020 | 9.c.1 Proportion of population covered by a mobile network, by technology |

| ***Goals and targets (from the 2030 Agenda for Sustainable Development)*** | ***Indicators*** |
| --- | --- |
| **Goal 10. Reduce inequality within and among countries** | |
| 10.1 By 2030, progressively achieve and sustain income growth of the bottom 40 per cent of the population at a rate higher than the national average | 10.1.1 Growth rates of household expenditure or income per capita among the bottom 40 per cent of the population and the total population |
| 10.2 By 2030, empower and promote the social, economic and political inclusion of all, irrespective of age, sex, disability, race, ethnicity, origin, religion or economic or other status | 10.2.1 Proportion of people living below 50 per cent of median income, by sex, age and persons with disabilities |
| 10.3 Ensure equal opportunity and reduce inequalities of outcome, including by eliminating discriminatory laws, policies and practices and promoting appropriate legislation, policies and action in this regard | 10.3.1 Proportion of population reporting having personally felt discriminated against or harassed in the previous 12 months on the basis of a ground of discrimination prohibited under international human rights law |
| 10.4 Adopt policies, especially fiscal, wage and social protection policies, and progressively achieve greater equality | 10.4.1 Labour share of GDP |
|  | 10.4.2 Redistributive impact of fiscal policy^4^ |
| 10.6 Ensure enhanced representation and voice for developing countries in decision-making in global international economic and financial institutions in order to deliver more effective, credible, accountable and legitimate institutions | 10.6.1 Proportion of members and voting rights of developing countries in international organizations |
| 10.7 Facilitate orderly, safe, regular and responsible migration and mobility of people, including through the implementation of planned and well-managed migration policies | 10.7.2 Number of countries with migration policies that facilitate orderly, safe, regular and responsible migration and mobility of people |
|  | 10.7.3 Number of people who died or disappeared in the process of migration towards an international destination |
|  | 10.7.4 Proportion of the population who are refugees, by country of origin |
| 10.b Encourage official development assistance and financial flows, including foreign direct investment, to States where the need is greatest, in particular least developed countries, African countries, small island developing States and landlocked developing countries, in accordance with their national plans and programmes | 10.b.1 Total resource flows for development, by recipient and donor countries and type of flow (e.g. official development assistance, foreign direct investment and other flows) |

| ***Goals and targets (from the 2030 Agenda for Sustainable Development)*** | ***Indicators*** |
| --- | --- |
| **Goal 11. Make cities and human settlements inclusive, safe, resilient and sustainable** | |
| 11.1 By 2030, ensure access for all to adequate, safe and affordable housing and basic services and upgrade slums | 11.1.1 Proportion of urban population living in slums, informal settlements or inadequate housing |
| 11.2 By 2030, provide access to safe, affordable, accessible and sustainable transport systems for all, improving road safety, notably by expanding public transport, with special attention to the needs of those in vulnerable situations, women, children, persons with disabilities and older persons | 11.2.1 Proportion of population that has convenient access to public transport, by sex, age and persons with disabilities |
| 11.3 By 2030, enhance inclusive and sustainable urbanization and capacity for participatory, integrated and sustainable human settlement planning and management in all countries | 11.3.2 Proportion of cities with a direct participation structure of civil society in urban planning and management that operate regularly and democratically |
| 11.5 By 2030, significantly reduce the number of deaths and the number of people affected and substantially decrease the direct economic losses relative to global gross domestic product caused by disasters, including water-related disasters, with a focus on protecting the poor and people in vulnerable situations | 11.5.1 Number of deaths, missing persons and directly affected persons attributed to disasters per 100,000 population |
|  | 11.5.2 Direct economic loss in relation to global GDP, damage to critical infrastructure and number of disruptions to basic services, attributed to disasters |
| 11.6 By 2030, reduce the adverse per capita environmental impact of cities, including by paying special attention to air quality and municipal and other waste management | 11.6.1 Proportion of municipal solid waste collected and managed in controlled facilities out of total municipal waste generated, by cities |
|  | 11.6.2 Annual mean levels of fine particulate matter (e.g. PM2.5 and PM10) in cities (population weighted) |
| 11.7 By 2030, provide universal access to safe, inclusive and accessible, green and public spaces, in particular for women and children, older persons and persons with disabilities | 11.7.1 Average share of the built-up area of cities that is open space for public use for all, by sex, age and persons with disabilities |
|  | 11.7.2 Proportion of persons victim of physical or sexual harassment, by sex, age, disability status and place of occurrence, in the previous 12 months |
| 11.a Support positive economic, social and environmental links between urban, peri-urban and rural areas by strengthening national and regional development planning | 11.a.1 Number of countries that have national urban policies or regional development plans that (*a*) respond to population dynamics; (*b*) ensure balanced territorial development; and (*c*) increase local fiscal space |
| 11.b By 2020, substantially increase the number of cities and human settlements adopting and implementing integrated policies and plans towards inclusion, resource efficiency, mitigation and adaptation to climate change, resilience to disasters, and develop and implement, in line with the Sendai Framework for Disaster Risk Reduction 2015–2030, holistic disaster risk management at all levels | 11.b.2 Proportion of local governments that adopt and implement local disaster risk reduction strategies in line with national disaster risk reduction strategies |

| ***Goals and targets (from the 2030 Agenda for Sustainable Development)*** | ***Indicators*** |
| --- | --- |
| **Goal 12. Ensure sustainable consumption and production patterns** | |
| 12.1 Implement the 10Year Framework of Programmes on Sustainable Consumption and Production Patterns, all countries taking action, with developed countries taking the lead, taking into account the development and capabilities of developing countries | 12.1.1 Number of countries developing, adopting or implementing policy instruments aimed at supporting the shift to sustainable consumption and production |
| 12.3 By 2030, halve per capita global food waste at the retail and consumer levels and reduce food losses along production and supply chains, including post-harvest losses | 12.3.1 (*a*) Food loss index and (*b*) food waste index |
| 12.4 By 2020, achieve the environmentally sound management of chemicals and all wastes throughout their life cycle, in accordance with agreed international frameworks, and significantly reduce their release to air, water and soil in order to minimize their adverse impacts on human health and the environment | 12.4.1 Number of parties to international multilateral environmental agreements on hazardous waste, and other chemicals that meet their commitments and obligations in transmitting information as required by each relevant agreement |
|  | 12.4.2 (*a*) Hazardous waste generated per capita; and (*b*) proportion of hazardous waste treated, by type of treatment |
| 12.5 By 2030, substantially reduce waste generation through prevention, reduction, recycling and reuse | 12.5.1 National recycling rate, tons of material recycled |
| 12.8 By 2030, ensure that people everywhere have the relevant information and awareness for sustainable development and lifestyles in harmony with nature | 12.8.1 Extent to which (i) global citizenship education and (ii) education for sustainable development are mainstreamed in (*a*) national education policies; (*b*) curricula; (*c*) teacher education; and (*d*) student assessment |
| 12.a Support developing countries to strengthen their scientific and technological capacity to move towards more sustainable patterns of consumption and production | 12.a.1 Installed renewable energy-generating capacity in developing countries (in watts per capita) |

| ***Goals and targets (from the 2030 Agenda for Sustainable Development)*** | ***Indicators*** |
| --- | --- |
| **Goal 13. Take urgent action to combat climate change and its impacts**^3^ | |
| 13.1 Strengthen resilience and adaptive capacity to climate-related hazards and natural disasters in all countries | 13.1.3 Proportion of local governments that adopt and implement local disaster risk reduction strategies in line with national disaster risk reduction strategies |
| 13.2 Integrate climate change measures into national policies, strategies and planning | 13.2.1 Number of countries with nationally determined contributions, long-term strategies, national adaptation plans and adaptation communications, as reported to the secretariat of the United Nations Framework Convention on Climate Change |
|  | 13.2.2 Total greenhouse gas emissions per year |
| 13.3 Improve education, awareness-raising and human and institutional capacity on climate change mitigation, adaptation, impact reduction and early warning | 13.3.1 Extent to which (i) global citizenship education and (ii) education for sustainable development are mainstreamed in (*a*) national education policies; (*b*) curricula; (*c*) teacher education; and (*d*) student assessment |

| ***Goals and targets (from the 2030 Agenda for Sustainable Development)*** | ***Indicators*** |
| --- | --- |
| **Goal 14. Conserve and sustainably use the oceans, seas and marine resources for sustainable development** | |
| 14.2 By 2020, sustainably manage and protect marine and coastal ecosystems to avoid significant adverse impacts, including by strengthening their resilience, and take action for their restoration in order to achieve healthy and productive oceans | 14.2.1 Number of countries using ecosystem-based approaches to managing marine areas |

| ***Goals and targets (from the 2030 Agenda for Sustainable Development)*** | ***Indicators*** |
| --- | --- |
| **Goal 15. Protect, restore and promote sustainable use of terrestrial ecosystems, sustainably manage forests, combat desertification, and halt and reverse land degradation and halt biodiversity loss** | |
| 15.1 By 2020, ensure the conservation, restoration and sustainable use of terrestrial and inland freshwater ecosystems and their services, in particular forests, wetlands, mountains and drylands, in line with obligations under international agreements | 15.1.1 Forest area as a proportion of total land area |
|  | 15.1.2 Proportion of important sites for terrestrial and freshwater biodiversity that are covered by protected areas, by ecosystem type |
| 15.2 By 2020, promote the implementation of sustainable management of all types of forests, halt deforestation, restore degraded forests and substantially increase afforestation and reforestation globally | 15.2.1 Progress towards sustainable forest management |
| 15.3 By 2030, combat desertification, restore degraded land and soil, including land affected by desertification, drought and floods, and strive to achieve a land degradation-neutral world | 15.3.1 Proportion of land that is degraded over total land area |
| 15.4 By 2030, ensure the conservation of mountain ecosystems, including their biodiversity, in order to enhance their capacity to provide benefits that are essential for sustainable development | 15.4.1 Coverage by protected areas of important sites for mountain biodiversity |
| 15.6 Promote fair and equitable sharing of the benefits arising from the utilization of genetic resources and promote appropriate access to such resources, as internationally agreed | 15.6.1 Number of countries that have adopted legislative, administrative and policy frameworks to ensure fair and equitable sharing of benefits |

| ***Goals and targets (from the 2030 Agenda for Sustainable Development)*** | ***Indicators*** |
| --- | --- |
| **Goal 16. Promote peaceful and inclusive societies for sustainable development, provide access to justice for all and build effective, accountable and inclusive institutions at all levels** | |
| 16.1 Significantly reduce all forms of violence and related death rates everywhere | 16.1.1 Number of victims of intentional homicide per 100,000 population, by sex and age |
|  | 16.1.2 Conflict-related deaths per 100,000 population, by sex, age and cause |
|  | 16.1.3 Proportion of population subjected to (*a*) physical violence, (*b*) psychological violence and (*c*) sexual violence in the previous 12 months |
|  | 16.1.4 Proportion of population that feel safe walking alone around the area they live |
| 16.2 End abuse, exploitation, trafficking and all forms of violence against and torture of children | 16.2.1 Proportion of children aged 1–17 years who experienced any physical punishment and/or psychological aggression by caregivers in the past month |
|  | 16.2.2 Number of victims of human trafficking per 100,000 population, by sex, age and form of exploitation |
|  | 16.2.3 Proportion of young women and men aged 18–29 years who experienced sexual violence by age 18 |
| 16.3 Promote the rule of law at the national and international levels and ensure equal access to justice for all | 16.3.1 Proportion of victims of violence in the previous 12 months who reported their victimization to competent authorities or other officially recognized conflict resolution mechanisms |
|  | 16.3.2 Unsentenced detainees as a proportion of overall prison population |
| 16.6 Develop effective, accountable and transparent institutions at all levels | 16.6.1 Primary government expenditures as a proportion of original approved budget, by sector (or by budget codes or similar) |
|  | 16.6.2 Proportion of population satisfied with their last experience of public services |
| 16.7 Ensure responsive, inclusive, participatory and representative decision-making at all levels | 16.7.1 Proportions of positions in national and local institutions, including (*a*) the legislatures; (*b*) the public service; and (*c*) the judiciary, compared to national distributions, by sex, age, persons with disabilities and population groups |
|  | 16.7.2 Proportion of population who believe decision-making is inclusive and responsive, by sex, age, disability and population group |
| 16.9 By 2030, provide legal identity for all, including birth registration | 16.9.1 Proportion of children under 5 years of age whose births have been registered with a civil authority, by age |
| 16.10 Ensure public access to information and protect fundamental freedoms, in accordance with national legislation and international agreements | 16.10.1 Number of verified cases of killing, kidnapping, enforced disappearance, arbitrary detention and torture of journalists, associated media personnel, trade unionists and human rights advocates in the previous 12 months |
|  | 16.10.2 Number of countries that adopt and implement constitutional, statutory and/or policy guarantees for public access to information |
| 16.a Strengthen relevant national institutions, including through international cooperation, for building capacity at all levels, in particular in developing countries, to prevent violence and combat terrorism and crime | 16.a.1 Existence of independent national human rights institutions in compliance with the Paris Principles |
| 16.b Promote and enforce non-discriminatory laws and policies for sustainable development | 16.b.1 Proportion of population reporting having personally felt discriminated against or harassed in the previous 12 months on the basis of a ground of discrimination prohibited under international human rights law |

| ***Goals and targets (from the 2030 Agenda for Sustainable Development)*** | ***Indicators*** | |
| --- | --- | --- |
| **Goal 17. Strengthen the means of implementation and revitalize the Global Partnership for Sustainable Development** | | |
| **Finance** | |  |
| 17.1 Strengthen domestic resource mobilization, including through international support to developing countries, to improve domestic capacity for tax and other revenue collection | | 17.1.1 Total government revenue as a proportion of GDP, by source |
| **Technology** | |  |
| 17.6 Enhance North-South, South-South and triangular regional and international cooperation on and access to science, technology and innovation and enhance knowledge-sharing on mutually agreed terms, including through improved coordination among existing mechanisms, in particular at the United Nations level, and through a global technology facilitation mechanism | | 17.6.1 Fixed Internet broadband subscriptions per 100 inhabitants, by speed^5^ |
| 17.8 Fully operationalize the technology bank and science, technology and innovation capacity-building mechanism for least developed countries by 2017 and enhance the use of enabling technology, in particular information and communications technology | | 17.8.1 Proportion of individuals using the Internet |
| **Systemic issues** | |  |
| *Policy and institutional coherence* | |  |
| 17.14 Enhance policy coherence for sustainable development | | 17.14.1 Number of countries with mechanisms in place to enhance policy coherence of sustainable development |
| *Multi-stakeholder partnerships* | |  |
| 17.16 Enhance the Global Partnership for Sustainable Development, complemented by multi-stakeholder partnerships that mobilize and share knowledge, expertise, technology and financial resources, to support the achievement of the Sustainable Development Goals in all countries, in particular developing countries | | 17.16.1 Number of countries reporting progress in multi-stakeholder development effectiveness monitoring frameworks that support the achievement of the sustainable development goals |
| *Data, monitoring and accountability* | |  |
| 17.18 By 2020, enhance capacity-building support to developing countries, including for least developed countries and small island developing States, to increase significantly the availability of high-quality, timely and reliable data disaggregated by income, gender, age, race, ethnicity, migratory status, disability, geographic location and other characteristics relevant in national contexts | | 17.18.1 Statistical capacity indicator for Sustainable Development Goal monitoring |
| 17.19 By 2030, build on existing initiatives to develop measurements of progress on sustainable development that complement gross domestic product, and support statistical capacity-building in developing countries | | 17.19.2 Proportion of countries that (*a*) have conducted at least one population and housing census in the last 10 years; and (*b*) have achieved 100 per cent birth registration and 80 per cent death registration |
