# Supplementary material for: Sustainable Development Goals as a Framework for Teaching and Learning about Health Equity in European Health and Social Care Study Programmes: A Modified Delphi Approach
Source: J Med Syst. 2025 Dec 22;49(1):187. doi: 10.1007/s10916-025-02328-3 (PMC12722463; doi:10.1007/s10916-025-02328-3)
Supplement: Supplementary file 2 — Supplementary Material 2 (DOCX 36.7 KB) [file 10916_2025_2328_MOESM2_ESM.docx]

**SUPPLEMENTARY FILE 2: RESULTS AFTER ROUND 1**

**Supplementary file 2** presents all the indicators included, excluded and re-rated after Round 1.

**Table S2.1.** 48 indicators were included after Round 1.

| *INDICATOR* | *MEAN* |
| --- | --- |
| 1.1.1 Proportion of the population living below the international poverty line by sex, age, employment status and geographic location (urban/rural) | **1,80** |
| 1.2.1 Proportion of population living below the national poverty line, by sex and age | **1,80** |
| 1.2.2 Proportion of men, women and children of all ages living in poverty in all its dimensions according to national definitions | **1,80** |
| 1.3.1 Proportion of population covered by social protection floors/systems, by sex, distinguishing children, unemployed persons, older persons, persons with disabilities, pregnant women, newborns, work-injury victims and the poor and the vulnerable | **1,89** |
| 1.4.1 Proportion of population living in households with access to basic services | **1,67** |
| 2.1.1 Prevalence of undernourishment | **1,70** |
| 2.1.2 Prevalence of moderate or severe food insecurity in the population, based on the Food Insecurity Experience Scale (FIES) | **1,80** |
| 2.2.2 Prevalence of malnutrition (weight for height >+2 or <-2 standard deviation from the median of the WHO Child Growth Standards) among children under 5 years of age, by type (wasting and overweight) | **1,70** |
| 3.1.1 Maternal mortality ratio | **2,00** |
| 3.1.2 Proportion of births attended by skilled health personnel | **1,80** |
| 3.2.1 Under5 mortality rate | **1,90** |
| 3.2.2 Neonatal mortality rate | **1,90** |
| 3.3.1 Number of new HIV infections per 1,000 uninfected population, by sex, age and key populations | **1,80** |
| 3.3.2 Tuberculosis incidence per 100,000 population | **1,70** |
| 3.3.4 Hepatitis B incidence per 100,000 population | **1,70** |
| 3.4.1 Mortality rate attributed to cardiovascular disease, cancer, diabetes or chronic respiratory disease | **1,80** |
| 3.4.2 Suicide mortality rate | **1,90** |
| 3.5.1 Coverage of treatment interventions (pharmacological, psychosocial and rehabilitation and aftercare services) for substance use disorders | **2,00** |
| 3.7.1 Proportion of women of reproductive age (aged 15–49 years) who have their need for family planning satisfied with modern methods | **1,70** |
| 3.7.2 Adolescent birth rate (aged 10–14 years; aged 15–19 years) per 1,000 women in that age group | **1,80** |
| 3.8.1 Coverage of essential health services | **1,90** |
| 3.9.1 Mortality rate attributed to household and ambient air pollution | **1,70** |
| 3.9.2 Mortality rate attributed to unsafe water, unsafe sanitation and lack of hygiene (exposure to unsafe Water, Sanitation and Hygiene for All (WASH) services) | **1,80** |
| 3.a.1 Age-standardized prevalence of current tobacco use among persons aged 15 years and older | **1,70** |
| 3.b.1 Proportion of the target population covered by all vaccines included in their national programme | **1,90** |
| 3.b.2 Total net official development assistance to medical research and basic health sectors | **1,70** |
| 3.b.3 Proportion of health facilities that have a core set of relevant essential medicines available and affordable on a sustainable basis | **1,90** |
| 3.c.1 Health worker density and distribution | **1,80** |
| 5.1.1 Whether or not legal frameworks are in place to promote, enforce and monitor equality and nondiscrimination on the basis of sex | **1,60** |
| 5.3.2 Proportion of girls and women aged 15–49 years who have undergone female genital mutilation/cutting, by age | **1,80** |
| 5.6.1 Proportion of women aged 15–49 years who make their own informed decisions regarding sexual relations, contraceptive use and reproductive health care | **1,80** |
| 6.1.1 Proportion of population using safely managed drinking water services | **1,80** |
| 6.2.1 Proportion of population using (*a*) safely managed sanitation services and (*b*) a hand-washing facility with soap and water | **2,00** |
| 8.5.1 Average hourly earnings of employees, by sex, age, occupation and persons with disabilities | **1,60** |
| 8.5.2 Unemployment rate, by sex, age and persons with disabilities | **1,70** |
| 8.8.1 Fatal and non-fatal occupational injuries per 100,000 workers, by sex and migrant status | **1,60** |
| 10.2.1 Proportion of people living below 50 per cent of median income, by sex, age and persons with disabilities | **1,70** |
| 10.3.1 Proportion of population reporting having personally felt discriminated against or harassed in the previous 12 months on the basis of a ground of discrimination prohibited under international human rights law | **1,70** |
| 10.7.2 Number of countries with migration policies that facilitate orderly, safe, regular and responsible migration and mobility of people | **1,60** |
| 10.7.4 Proportion of the population who are refugees, by country of origin | **1,60** |
| 11.1.1 Proportion of urban population living in slums, informal settlements or inadequate housing | **1,80** |
| 11.2.1 Proportion of population that has convenient access to public transport, by sex, age and persons with disabilities | **1,60** |
| 11.7.1 Average share of the built-up area of cities that is open space for public use for all, by sex, age and persons with disabilities | **1,70** |
| 11.7.2 Proportion of persons victim of physical or sexual harassment, by sex, age, disability status and place of occurrence, in the previous 12 months | **1,70** |
| 16.1.3 Proportion of population subjected to (*a*) physical violence, (*b*) psychological violence and (*c*) sexual violence in the previous 12 months | **1,70** |
| 16.2.1 Proportion of children aged 1–17 years who experienced any physical punishment and/or psychological aggression by caregivers in the past month | **1,60** |
| 16.2.3 Proportion of young women and men aged 18–29 years who experienced sexual violence by age 18 | **1,60** |
| 16.b.1 Proportion of population reporting having personally felt discriminated against or harassed in the previous 12 months on the basis of a ground of discrimination prohibited under international human rights law | **1,60** |

**Table S2.2.** 60 indicators were excluded after Round 1.

| *INDICATOR* | *MEAN* |
| --- | --- |
| 2.3.1 Volume of production per labour unit by classes of farming/pastoral/forestry enterprise size | **0,20** |
| 2.5.1 Number of (*a*) plant and (*b*) animal genetic resources for food and agriculture secured in either medium- or long-term conservation facilities | **0,30** |
| 2.5.2 Proportion of local breeds classified as being at risk of extinction | **0,30** |
| 2.a.1 The agriculture orientation index for government expenditures | **0,30** |
| 2.b.1 Agricultural export subsidies | **0,20** |
| 5.a.1 (*a*) Proportion of total agricultural population with ownership or secure rights over agricultural land, by sex; and (*b*) share of women among owners or rights-bearers of agricultural land, by type of tenure | **0,40** |
| 8.4.1 Material footprint, material footprint per capita, and material footprint per GDP | **0,30** |
| 8.4.2 Domestic material consumption, domestic material consumption per capita, and domestic material consumption per GDP | **0,40** |
| 8.9.1 Tourism direct GDP as a proportion of total GDP and in growth rate | **0,30** |
| 8.10.1 (*a*) Number of commercial bank branches per 100,000 adults and (*b*) number of automated teller machines (ATMs) per 100,000 adults | **0,20** |
| 8.10.2 Proportion of adults (15 years and older) with an account at a bank or other financial institution or with a mobile-money-service provider | **0,30** |
| 8.a.1 Aid for Trade commitments and disbursements | **0,30** |
| 9.1.2 Passenger and freight volumes, by mode of transport | **0,10** |
| 9.2.1 Manufacturing value added as a proportion of GDP and per capita | **0,10** |
| 9.2.2 Manufacturing employment as a proportion of total employment | **0,30** |
| 9.3.1 Proportion of small-scale industries in total industry value added | **0,10** |
| 9.3.2 Proportion of small-scale industries with a loan or line of credit | **0,20** |
| 9.5.1 Research and development expenditure as a proportion of GDP | **0,40** |
| 9.b.1 Proportion of medium and high-tech industry value added in total value added | **0,30** |
| 10.5.1 Financial Soundness Indicators | **0,40** |
| 10.7.1 Recruitment cost borne by employee as a proportion of monthly income earned in country of destination | **0,10** |
| 10.a.1 Proportion of tariff lines applied to imports from least developed countries and developing countries with zero-tariff | **0,40** |
| 10.c.1 Remittance costs as a proportion of the amount remitted | **0,20** |
| 11.3.1 Ratio of land consumption rate to population growth rate | **0,40** |
| 12.2.1 Material footprint, material footprint per capita, and material footprint per GDP | **0,40** |
| 12.2.2 Domestic material consumption, domestic material consumption per capita, and domestic material consumption per GDP | **0,40** |
| 12.c.1 Amount of fossil-fuel subsidies (production and consumption) per unit of GDP | **0,30** |
| 13.a.1 Amounts provided and mobilized in United States dollars per year in relation to the continued existing collective mobilization goal of the $100 billion commitment through to 2025 | **0,20** |
| 13.b.1 Number of least developed countries and small island developing States with nationally determined contributions, long-term strategies, national adaptation plans and adaptation communications, as reported to the secretariat of the United Nations Framework Convention on Climate Change | **0,20** |
| 14.1.1 (*a*) Index of coastal eutrophication; and (*b*) plastic debris density | **0,40** |
| 14.3.1 Average marine acidity (pH) measured at agreed suite of representative sampling stations | **0,40** |
| 14.4.1 Proportion of fish stocks within biologically sustainable levels | **0,40** |
| 14.5.1 Coverage of protected areas in relation to marine areas | **0,20** |
| 14.6.1 Degree of implementation of international instruments aiming to combat illegal, unreported and unregulated fishing | **0,20** |
| 14.7.1 Sustainable fisheries as a proportion of GDP in small island developing States, least developed countries and all countries | **0,20** |
| 14.a.1 Proportion of total research budget allocated to research in the field of marine technology | **0,00** |
| 14.b.1 Degree of application of a legal/regulatory/ policy/institutional framework which recognizes and protects access rights for small-scale fisheries | **0,30** |
| 14.c.1 Number of countries making progress in ratifying, accepting and implementing through legal, policy and institutional frameworks, ocean-related instruments that implement international law, as reflected in the United Nations Convention on the Law of the Sea, for the conservation and sustainable use of the oceans and their resources | **0,30** |
| 15.4.2 Mountain Green Cover Index | **0,40** |
| 15.7.1 Proportion of traded wildlife that was poached or illicitly trafficked | **0,10** |
| 15.8.1 Proportion of countries adopting relevant national legislation and adequately resourcing the prevention or control of invasive alien species | **0,30** |
| 15.9.1 (*a*) Number of countries that have established national targets in accordance with or similar to Aichi Biodiversity Target 2 of the Strategic Plan for Biodiversity 2011–2020 in their national biodiversity strategy and action plans and the progress reported towards these targets; and (*b*) integration of biodiversity into national accounting and reporting systems, defined as implementation of the System of Environmental-Economic Accounting | **0,30** |
| 15.a.1 (*a*) Official development assistance on conservation and sustainable use of biodiversity; and (*b*) revenue generated and finance mobilized from biodiversity-relevant economic instruments | **0,30** |
| 15.b.1 (*a*) Official development assistance on conservation and sustainable use of biodiversity; and (*b*) revenue generated and finance mobilized from biodiversity-relevant economic instruments | **0,30** |
| 15.c.1 Proportion of traded wildlife that was poached or illicitly trafficked | **0,30** |
| 16.3.3 Proportion of the population who have experienced a dispute in the past two years and who accessed a formal or informal dispute resolution mechanism, by type of mechanism | **0,40** |
| 16.4.1 Total value of inward and outward illicit financial flows (in current United States dollars) | **0,30** |
| 16.4.2 Proportion of seized, found or surrendered arms whose illicit origin or context has been traced or established by a competent authority in line with international instruments | **0,30** |
| 17.2.1 Net official development assistance, total and to least developed countries, as a proportion of the Organization for Economic Cooperation and Development (OECD) Development Assistance Committee donors’ gross national income (GNI) | **0,40** |
| 17.3.1 Foreign direct investment, official development assistance and South-South cooperation as a proportion of gross national income | **0,40** |
| 17.7.1 Total amount of funding for developing countries to promote the development, transfer, dissemination and diffusion of environmentally sound technologies | **0,40** |
| 17.9.1 Dollar value of financial and technical assistance (including through North-South, South-South and triangular cooperation) committed to developing countries | **0,40** |
| 17.10.1 Worldwide weighted tariff-average | **0,40** |
| 17.11.1 Developing countries’ and least developed countries’ share of global exports | **0,30** |
| 17.12.1 Weighted average tariffs faced by developing countries, least developed countries and small island developing States | **0,40** |
| 17.15.1 Extent of use of country-owned results frameworks and planning tools by providers of development cooperation | **0,40** |
| 17.17.1 Amount in United States dollars committed to public-private partnerships for infrastructure | **0,20** |
| 17.18.2 Number of countries that have national statistical legislation that complies with the Fundamental Principles of Official Statistics | **0,40** |
| 17.18.3 Number of countries with a national statistical plan that is fully funded and under implementation, by source of funding | **0,10** |
| 17.19.1 Dollar value of all resources made available to strengthen statistical capacity in developing countries | **0,10** |

**Table S2.3.** 139 indicators were re-rated after Round 1.

| *INDICATORS* | *MEAN* |
| --- | --- |
| 1.4.2 Proportion of total adult population with secure tenure rights to land, (*a*) with legally recognized documentation, and (*b*) who perceive their rights to land as secure, by sex and type of tenure | **0,89** |
| 1.5.1 Number of deaths, missing persons and directly affected persons attributed to disasters per 100,000 population | **1,00** |
| 1.5.2 Direct economic loss attributed to disasters in relation to global gross domestic product (GDP) | **1,00** |
| 1.5.3 Number of countries that adopt and implement national disaster risk reduction strategies in line with the Sendai Framework for Disaster Risk Reduction 2015–2030 | **0,90** |
| 1.5.4 Proportion of local governments that adopt and implement local disaster risk reduction strategies in line with national disaster risk reduction strategies | **0,90** |
| 1.a.1 Total official development assistance grants from all donors that focus on poverty reduction as a share of the recipient country’s gross national income | **1,10** |
| 1.a.2 Proportion of total government spending on essential services (education, health and social protection) | **1,50** |
| 1.b.1 Pro-poor public social spending | **1,50** |
| 2.2.1 Prevalence of stunting (height for age <-2 standard deviation from the median of the World Health Organization (WHO) Child Growth Standards) among children under 5 years of age | **1,30** |
| 2.2.3 Prevalence of anaemia in women aged 15 to 49 years, by pregnancy status (percentage) | **1,40** |
| 2.3.2 Average income of small-scale food producers, by sex and indigenous status | **0,70** |
| 2.4.1 Proportion of agricultural area under productive and sustainable agriculture | **0,70** |
| 2.a.2 Total official flows (official development assistance plus other official flows) to the agriculture sector | **0,60** |
| 2.c.1 Indicator of food price anomalies | **0,90** |
| 3.3.3 Malaria incidence per 1,000 population | **1,40** |
| 3.3.5 Number of people requiring interventions against neglected tropical diseases | **1,30** |
| 3.5.2 Alcohol per capita consumption (aged 15 years and older) within a calendar year in litres of pure alcohol | **1,40** |
| 3.6.1 Death rate due to road traffic injuries | **1,30** |
| 3.8.2 Proportion of population with large household expenditures on health as a share of total household expenditure or income | **1,50** |
| 3.9.3 Mortality rate attributed to unintentional poisoning | **1,30** |
| 3.d.1 International Health Regulations (IHR) capacity and health emergency preparedness | **1,50** |
| 3.d.2 Percentage of bloodstream infections due to selected antimicrobial-resistant organisms | **1,30** |
| 4.1.1 Proportion of children and young people (*a*) in grades 2/3; (*b*) at the end of primary; and (*c*) at the end of lower secondary achieving at least a minimum proficiency level in (i) reading and (ii) mathematics, by sex | **1,20** |
| 4.1.2 Completion rate (primary education, lower secondary education, upper secondary education) | **1,30** |
| 4.2.1 Proportion of children aged 24–59 months who are developmentally on track in health, learning and psychosocial well-being, by sex | **1,50** |
| 4.2.2 Participation rate in organized learning (one year before the official primary entry age), by sex | **0,80** |
| 4.3.1 Participation rate of youth and adults in formal and non-formal education and training in the previous 12 months, by sex | **1,40** |
| 4.4.1 Proportion of youth and adults with information and communications technology (ICT) skills, by type of skill | **1,20** |
| 4.5.1 Parity indices (female/male, rural/urban, bottom/top wealth quintile and others such as disability status, indigenous peoples and conflict-affected, as data become available) for all education indicators on this list that can be disaggregated | **1,50** |
| 4.6.1 Proportion of population in a given age group achieving at least a fixed level of proficiency in functional (*a*) literacy and (*b*) numeracy skills, by sex | **1,10** |
| 4.7.1 Extent to which (i) global citizenship education and (ii) education for sustainable development are mainstreamed in (*a*) national education policies; (*b*) curricula; (*c*) teacher education; and (*d*) student assessment | **1,20** |
| 4.a.1 Proportion of schools offering basic services, by type of service | **0,90** |
| 4.b.1 Volume of official development assistance flows for scholarships by sector and type of study | **0,50** |
| 4.c.1 Proportion of teachers with the minimum required qualifications, by education level | **0,60** |
| 5.2.1 Proportion of ever-partnered women and girls aged 15 years and older subjected to physical, sexual or psychological violence by a current or former intimate partner in the previous 12 months, by form of violence and by age | **1,40** |
| 5.2.2 Proportion of women and girls aged 15 years and older subjected to sexual violence by persons other than an intimate partner in the previous 12 months, by age and place of occurrence | **1,50** |
| 5.3.1 Proportion of women aged 20–24 years who were married or in a union before age 15 and before age 18 | **1,40** |
| 5.4.1 Proportion of time spent on unpaid domestic and care work, by sex, age and location | **1,40** |
| 5.5.1 Proportion of seats held by women in (*a*) national parliaments and (*b*) local governments | **1,10** |
| 5.5.2 Proportion of women in managerial positions | **1,10** |
| 5.6.2 Number of countries with laws and regulations that guarantee full and equal access to women and men aged 15 years and older to sexual and reproductive health care, information and education | **1,40** |
| 5.a.2 Proportion of countries where the legal framework (including customary law) guarantees women’s equal rights to land ownership and/or control | **0,60** |
| 5.b.1 Proportion of individuals who own a mobile telephone, by sex | **0,80** |
| 5.c.1 Proportion of countries with systems to track and make public allocations for gender equality and women’s empowerment | **1,10** |
| 6.3.1 Proportion of domestic and industrial wastewater flows safely treated | **1,10** |
| 6.3.2 Proportion of bodies of water with good ambient water quality | **1,30** |
| 6.4.1 Change in water-use efficiency over time | **0,70** |
| 6.4.2 Level of water stress: freshwater withdrawal as a proportion of available freshwater resources | **1,10** |
| 6.5.1 Degree of integrated water resources management | **0,50** |
| 6.5.2 Proportion of transboundary basin area with an operational arrangement for water cooperation | **0,50** |
| 6.6.1 Change in the extent of water-related ecosystems over time | **1,10** |
| 6.a.1 Amount of water- and sanitation-related official development assistance that is part of a government-coordinated spending plan | **0,50** |
| 6.b.1 Proportion of local administrative units with established and operational policies and procedures for participation of local communities in water and sanitation management | **0,70** |
| 7.1.1 Proportion of population with access to electricity | **1,40** |
| 7.1.2 Proportion of population with primary reliance on clean fuels and technology | **1,10** |
| 7.2.1 Renewable energy share in the total final energy consumption | **0,90** |
| 7.3.1 Energy intensity measured in terms of primary energy and GDP | **0,50** |
| 7.a.1 International financial flows to developing countries in support of clean energy research and development and renewable energy production, including in hybrid systems | **0,80** |
| 7.b.1 Installed renewable energy-generating capacity in developing countries (in watts per capita) | **0,70** |
| 8.1.1 Annual growth rate of real GDP per capita | **0,80** |
| 8.2.1 Annual growth rate of real GDP per employed person | **0,80** |
| 8.3.1 Proportion of informal employment in total employment, by sector and sex | **1,20** |
| 8.6.1 Proportion of youth (aged 15–24 years) not in education, employment or training | **1,30** |
| 8.7.1 Proportion and number of children aged 5–17 years engaged in child labour, by sex and age | **1,50** |
| 8.8.2 Level of national compliance with labour rights (freedom of association and collective bargaining) based on International Labour Organization (ILO) textual sources and national legislation, by sex and migrant status | **1,20** |
| 8.b.1 Existence of a developed and operationalized national strategy for youth employment, as a distinct strategy or as part of a national employment strategy | **1,00** |
| 9.1.1 Proportion of the rural population who live within 2 km of an all-season road | **1,00** |
| 9.4.1 CO_2_ emission per unit of value added | **0,90** |
| 9.5.2 Researchers (in full-time equivalent) per million inhabitants | **0,70** |
| 9.a.1 Total official international support (official development assistance plus other official flows) to infrastructure | **0,70** |
| 9.c.1 Proportion of population covered by a mobile network, by technology | **1,10** |
| 10.1.1 Growth rates of household expenditure or income per capita among the bottom 40 per cent of the population and the total population | **1,30** |
| 10.4.1 Labour share of GDP | **0,90** |
| 10.4.2 Redistributive impact of fiscal policy^4^ | **0,50** |
| 10.6.1 Proportion of members and voting rights of developing countries in international organizations | **0,80** |
| 10.7.3 Number of people who died or disappeared in the process of migration towards an international destination | **1,40** |
| 10.b.1 Total resource flows for development, by recipient and donor countries and type of flow (e.g. official development assistance, foreign direct investment and other flows) | **0,70** |
| 11.3.2 Proportion of cities with a direct participation structure of civil society in urban planning and management that operate regularly and democratically | **0,60** |
| 11.4.1 Total per capita expenditure on the preservation, protection and conservation of all cultural and natural heritage, by source of funding (public, private), type of heritage (cultural, natural) and level of government (national, regional, and local/municipal) | **0,60** |
| 11.5.1 Number of deaths, missing persons and directly affected persons attributed to disasters per 100,000 population | **1,30** |
| 11.5.2 Direct economic loss in relation to global GDP, damage to critical infrastructure and number of disruptions to basic services, attributed to disasters | **1,10** |
| 11.6.1 Proportion of municipal solid waste collected and managed in controlled facilities out of total municipal waste generated, by cities | **0,90** |
| 11.6.2 Annual mean levels of fine particulate matter (e.g. PM2.5 and PM10) in cities (population weighted) | **0,70** |
| 11.a.1 Number of countries that have national urban policies or regional development plans that (*a*) respond to population dynamics; (*b*) ensure balanced territorial development; and (*c*) increase local fiscal space | **0,60** |
| 11.b.1 Number of countries that adopt and implement national disaster risk reduction strategies in line with the Sendai Framework for Disaster Risk Reduction 2015–2030 | **1,00** |
| 11.b.2 Proportion of local governments that adopt and implement local disaster risk reduction strategies in line with national disaster risk reduction strategies | **1,10** |
| 12.1.1 Number of countries developing, adopting or implementing policy instruments aimed at supporting the shift to sustainable consumption and production | **0,80** |
| 12.3.1 (*a*) Food loss index and (*b*) food waste index | **1,00** |
| 12.4.1 Number of parties to international multilateral environmental agreements on hazardous waste, and other chemicals that meet their commitments and obligations in transmitting information as required by each relevant agreement | **0,80** |
| 12.4.2 (*a*) Hazardous waste generated per capita; and (*b*) proportion of hazardous waste treated, by type of treatment | **0,90** |
| 12.5.1 National recycling rate, tons of material recycled | **1,00** |
| 12.6.1 Number of companies publishing sustainability reports | **0,70** |
| 12.7.1 Degree of sustainable public procurement policies and action plan implementation | **0,70** |
| 12.8.1 Extent to which (i) global citizenship education and (ii) education for sustainable development are mainstreamed in (*a*) national education policies; (*b*) curricula; (*c*) teacher education; and (*d*) student assessment | **1,20** |
| 12.a.1 Installed renewable energy-generating capacity in developing countries (in watts per capita) | **0,50** |
| 12.b.1 Implementation of standard accounting tools to monitor the economic and environmental aspects of tourism sustainability | **0,50** |
| 13.1.1 Number of deaths, missing persons and directly affected persons attributed to disasters per 100,000 population | **1,30** |
| 13.1.2 Number of countries that adopt and implement national disaster risk reduction strategies in line with the Sendai Framework for Disaster Risk Reduction 2015–2030 | **1,10** |
| 13.1.3 Proportion of local governments that adopt and implement local disaster risk reduction strategies in line with national disaster risk reduction strategies | **1,10** |
| 13.2.1 Number of countries with nationally determined contributions, long-term strategies, national adaptation plans and adaptation communications, as reported to the secretariat of the United Nations Framework Convention on Climate Change | **1,00** |
| 13.2.2 Total greenhouse gas emissions per year | **1,10** |
| 13.3.1 Extent to which (i) global citizenship education and (ii) education for sustainable development are mainstreamed in (*a*) national education policies; (*b*) curricula; (*c*) teacher education; and (*d*) student assessment | **1,30** |
| 14.2.1 Number of countries using ecosystem-based approaches to managing marine areas | **0,80** |
| 15.1.1 Forest area as a proportion of total land area | **0,70** |
| 15.1.2 Proportion of important sites for terrestrial and freshwater biodiversity that are covered by protected areas, by ecosystem type | **0,70** |
| 15.2.1 Progress towards sustainable forest management | **0,60** |
| 15.3.1 Proportion of land that is degraded over total land area | **0,80** |
| 15.4.1 Coverage by protected areas of important sites for mountain biodiversity | **0,60** |
| 15.5.1 Red List Index | **0,60** |
| 15.6.1 Number of countries that have adopted legislative, administrative and policy frameworks to ensure fair and equitable sharing of benefits | **1,00** |
| 16.1.1 Number of victims of intentional homicide per 100,000 population, by sex and age | **1,50** |
| 16.1.2 Conflict-related deaths per 100,000 population, by sex, age and cause | **1,40** |
| 16.1.4 Proportion of population that feel safe walking alone around the area they live | **1,50** |
| 16.2.2 Number of victims of human trafficking per 100,000 population, by sex, age and form of exploitation | **1,50** |
| 16.3.1 Proportion of victims of violence in the previous 12 months who reported their victimization to competent authorities or other officially recognized conflict resolution mechanisms | **1,40** |
| 16.3.2 Unsentenced detainees as a proportion of overall prison population | **1,00** |
| 16.5.1 Proportion of persons who had at least one contact with a public official and who paid a bribe to a public official, or were asked for a bribe by those public officials, during the previous 12 months | **0,70** |
| 16.5.2 Proportion of businesses that had at least one contact with a public official and that paid a bribe to a public official, or were asked for a bribe by those public officials during the previous 12 months | **0,70** |
| 16.6.1 Primary government expenditures as a proportion of original approved budget, by sector (or by budget codes or similar) | **0,60** |
| 16.6.2 Proportion of population satisfied with their last experience of public services | **1,20** |
| 16.7.1 Proportions of positions in national and local institutions, including (*a*) the legislatures; (*b*) the public service; and (*c*) the judiciary, compared to national distributions, by sex, age, persons with disabilities and population groups | **1,00** |
| 16.7.2 Proportion of population who believe decision-making is inclusive and responsive, by sex, age, disability and population group | **1,40** |
| 16.8.1 Proportion of members and voting rights of developing countries in international organizations | **0,90** |
| 16.9.1 Proportion of children under 5 years of age whose births have been registered with a civil authority, by age | **1,10** |
| 16.10.1 Number of verified cases of killing, kidnapping, enforced disappearance, arbitrary detention and torture of journalists, associated media personnel, trade unionists and human rights advocates in the previous 12 months | **1,30** |
| 16.10.2 Number of countries that adopt and implement constitutional, statutory and/or policy guarantees for public access to information | **1,10** |
| 16.a.1 Existence of independent national human rights institutions in compliance with the Paris Principles | **1,20** |
| 17.1.1 Total government revenue as a proportion of GDP, by source | **0,80** |
| 17.1.2 Proportion of domestic budget funded by domestic taxes | **0,70** |
| 17.3.2 Volume of remittances (in United States dollars) as a proportion of total GDP | **0,60** |
| 17.4.1 Debt service as a proportion of exports of goods and services | **0,60** |
| 17.5.1 Number of countries that adopt and implement investment promotion regimes for developing countries, including the least developed countries | **0,50** |
| 17.6.1 Fixed Internet broadband subscriptions per 100 inhabitants, by speed^5^ | **0,90** |
| 17.8.1 Proportion of individuals using the Internet | **1,20** |
| 17.13.1 Macroeconomic Dashboard | **0,50** |
| 17.14.1 Number of countries with mechanisms in place to enhance policy coherence of sustainable development | **0,60** |
| 17.16.1 Number of countries reporting progress in multi-stakeholder development effectiveness monitoring frameworks that support the achievement of the sustainable development goals | **0,60** |
| 17.18.1 Statistical capacity indicator for Sustainable Development Goal monitoring | **0,80** |
| 17.19.2 Proportion of countries that (*a*) have conducted at least one population and housing census in the last 10 years; and (*b*) have achieved 100 per cent birth registration and 80 per cent death registration | **0,80** |
