# Supplementary material for: Sustainable Development Goals as a Framework for Teaching and Learning about Health Equity in European Health and Social Care Study Programmes: A Modified Delphi Approach
Source: J Med Syst. 2025 Dec 22;49(1):187. doi: 10.1007/s10916-025-02328-3 (PMC12722463; doi:10.1007/s10916-025-02328-3)
Supplement: Supplementary file 3 — Supplementary Material 3 (DOCX 26.5 KB) [file 10916_2025_2328_MOESM3_ESM.docx]

**SUPPLEMENTARY FILE 3. RESULTS AFTER ROUND 2**

**Supplementary file 3** presents all the indicators included, excluded and re-rated after Round 2.

**Table S3.1.** 64 indicators were included after Round 2.

| *INDICATORS* | *MEAN* |
| --- | --- |
| 1.4.2 Proportion of total adult population with secure tenure rights to land, (a) with legally recognized documentation, and (b) who perceive their rights to land as secure, by sex and type of tenure | **1,57** |
| 1.a.2 Proportion of total government spending on essential services (education, health and social protection) | **2,00** |
| 1.b.1 Pro-poor public social spending | **2,00** |
| 2.2.1 Prevalence of stunting (height for age <-2 standard deviation from the median of the World Health Organization (WHO) Child Growth Standards) among children under 5 years of age | **2,00** |
| 2.2.3 Prevalence of anaemia in women aged 15 to 49 years, by pregnancy status (percentage) | **2,00** |
| 2.3.2 Average income of small-scale food producers, by sex and indigenous status | **2,00** |
| 3.3.5 Number of people requiring interventions against neglected tropical diseases | **1,67** |
| 3.5.2 Alcohol per capita consumption (aged 15 years and older) within a calendar year in litres of pure alcohol | **2,00** |
| 3.8.2 Proportion of population with large household expenditures on health as a share of total household expenditure or income | **2,00** |
| 3.d.1 International Health Regulations (IHR) capacity and health emergency preparedness | **2,00** |
| 4.1.1 Proportion of children and young people (a) in grades 2/3; (b) at the end of primary; and (c) at the end of lower secondary achieving at least a minimum proficiency level in (i) reading and (ii) mathematics, by sex | **2,00** |
| 4.2.2 Participation rate in organized learning (one year before the official primary entry age), by sex | **1,67** |
| 4.4.1 Proportion of youth and adults with information and communications technology (ICT) skills, by type of skill | **2,00** |
| 4.5.1 Parity indices (female/male, rural/urban, bottom/top wealth quintile and others such as disability status, indigenous peoples and conflict-affected, as data become available) for all education indicators on this list that can be disaggregated | **2,00** |
| 4.6.1 Proportion of population in a given age group achieving at least a fixed level of proficiency in functional (a) literacy and (b) numeracy skills, by sex | **1,67** |
| 4.7.1 Extent to which (i) global citizenship education and (ii) education for sustainable development are mainstreamed in (a) national education policies; (b) curricula; (c) teacher education; and (d) student assessment | **2,00** |
| 4.a.1 Proportion of schools offering basic services, by type of service | **1,67** |
| 5.2.1 Proportion of ever-partnered women and girls aged 15 years and older subjected to physical, sexual or psychological violence by a current or former intimate partner in the previous 12 months, by form of violence and by age | **2,00** |
| 5.2.2 Proportion of women and girls aged 15 years and older subjected to sexual violence by persons other than an intimate partner in the previous 12 months, by age and place of occurrence | **2,00** |
| 5.4.1 Proportion of time spent on unpaid domestic and care work, by sex, age and location | **2,00** |
| 5.5.1 Proportion of seats held by women in (a) national parliaments and (b) local governments | **2,00** |
| 5.6.2 Number of countries with laws and regulations that guarantee full and equal access to women and men aged 15 years and older to sexual and reproductive health care, information and education | **2,00** |
| 5.a.2 Proportion of countries where the legal framework (including customary law) guarantees women’s equal rights to land ownership and/or control | **1,67** |
| 5.b.1 Proportion of individuals who own a mobile telephone, by sex | **2,00** |
| 6.3.1 Proportion of domestic and industrial wastewater flows safely treated | **2,00** |
| 6.b.1 Proportion of local administrative units with established and operational policies and procedures for participation of local communities in water and sanitation management | **2,00** |
| 7.1.1 Proportion of population with access to electricity | **1,67** |
| 7.1.2 Proportion of population with primary reliance on clean fuels and technology | **2,00** |
| 8.3.1 Proportion of informal employment in total employment, by sector and sex | **2,00** |
| 8.6.1 Proportion of youth (aged 15–24 years) not in education, employment or training | **2,00** |
| 8.7.1 Proportion and number of children aged 5–17 years engaged in child labour, by sex and age | **1,67** |
| 9.1.1 Proportion of the rural population who live within 2 km of an all-season road | **2,00** |
| 9.5.2 Researchers (in full-time equivalent) per million inhabitants | **2,00** |
| 9.c.1 Proportion of population covered by a mobile network, by technology | **2,00** |
| 10.1.1 Growth rates of household expenditure or income per capita among the bottom 40 per cent of the population and the total population | **2,00** |
| 10.4.2 Redistributive impact of fiscal policy4 | **2,00** |
| 11.3.2 Proportion of cities with a direct participation structure of civil society in urban planning and management that operate regularly and democratically | **2,00** |
| 11.5.1 Number of deaths, missing persons and directly affected persons attributed to disasters per 100,000 population | **1,67** |
| 11.6.1 Proportion of municipal solid waste collected and managed in controlled facilities out of total municipal waste generated, by cities | **2,00** |
| 11.6.2 Annual mean levels of fine particulate matter (e.g. PM2.5 and PM10) in cities (population weighted) | **2,00** |
| 11.b.2 Proportion of local governments that adopt and implement local disaster risk reduction strategies in line with national disaster risk reduction strategies | **1,67** |
| 12.3.1 (a) Food loss index and (b) food waste index | **2,00** |
| 13.1.3 Proportion of local governments that adopt and implement local disaster risk reduction strategies in line with national disaster risk reduction strategies | **2,00** |
| 13.2.1 Number of countries with nationally determined contributions, long-term strategies, national adaptation plans and adaptation communications, as reported to the secretariat of the United Nations Framework Convention on Climate Change | **2,00** |
| 13.2.2 Total greenhouse gas emissions per year | **2,00** |
| 15.1.1 Forest area as a proportion of total land area | **1,67** |
| 15.2.1 Progress towards sustainable forest management | **1,67** |
| 15.6.1 Number of countries that have adopted legislative, administrative and policy frameworks to ensure fair and equitable sharing of benefits | **2,00** |
| 16.1.2 Conflict-related deaths per 100,000 population, by sex, age and cause | **1,67** |
| 16.2.2 Number of victims of human trafficking per 100,000 population, by sex, age and form of exploitation | **2,00** |
| 16.3.1 Proportion of victims of violence in the previous 12 months who reported their victimization to competent authorities or other officially recognized conflict resolution mechanisms | **2,00** |
| 16.3.2 Unsentenced detainees as a proportion of overall prison population | **2,00** |
| 16.6.1 Primary government expenditures as a proportion of original approved budget, by sector (or by budget codes or similar) | **2,00** |
| 16.6.2 Proportion of population satisfied with their last experience of public services | **2,00** |
| 16.7.1 Proportions of positions in national and local institutions, including (a) the legislatures; (b) the public service; and (c) the judiciary, compared to national distributions, by sex, age, persons with disabilities and population groups | **2,00** |
| 16.7.2 Proportion of population who believe decision-making is inclusive and responsive, by sex, age, disability and population group | **1,67** |
| 16.9.1 Proportion of children under 5 years of age whose births have been registered with a civil authority, by age | **1,67** |
| 16.10.1 Number of verified cases of killing, kidnapping, enforced disappearance, arbitrary detention and torture of journalists, associated media personnel, trade unionists and human rights advocates in the previous 12 months | **2,00** |
| 16.10.2 Number of countries that adopt and implement constitutional, statutory and/or policy guarantees for public access to information | **2,00** |
| 16.a.1 Existence of independent national human rights institutions in compliance with the Paris Principles | **2,00** |
| 17.8.1 Proportion of individuals using the Internet | **2,00** |
| 17.14.1 Number of countries with mechanisms in place to enhance policy coherence of sustainable development | **2,00** |
| 17.16.1 Number of countries reporting progress in multi-stakeholder development effectiveness monitoring frameworks that support the achievement of the sustainable development goals | **2,00** |
| 17.19.2 Proportion of countries that (a) have conducted at least one population and housing census in the last 10 years; and (b) have achieved 100 per cent birth registration and 80 per cent death registration | **2,00** |

**Table S.3.2.** 9 indicators were excluded after Round 2.

| *INDICATORS* | *MEAN* |
| --- | --- |
| 3.9.3 Mortality rate attributed to unintentional poisoning | **0,00** |
| 6.6.1 Change in the extent of water-related ecosystems over time | **0,00** |
| 7.3.1 Energy intensity measured in terms of primary energy and GDP | **0,00** |
| 12.6.1 Number of companies publishing sustainability reports | **0,33** |
| 12.7.1 Degree of sustainable public procurement policies and action plan implementation | **0,00** |
| 13.1.1 Number of deaths, missing persons and directly affected persons attributed to disasters per 100,000 population | **0,00** |
| 17.1.2 Proportion of domestic budget funded by domestic taxes | **0,00** |
| 17.4.1 Debt service as a proportion of exports of goods and services | **0,00** |
| 17.5.1 Number of countries that adopt and implement investment promotion regimes for developing countries, including the least developed countries | **0,33** |

**Table S.3.3.** 66 indicators were re-rated after Round 2.

| *INDICATORS* | *MEAN* |
| --- | --- |
| 1.5.1 Number of deaths, missing persons and directly affected persons attributed to disasters per 100,000 population | **1,33** |
| 1.5.2 Direct economic loss attributed to disasters in relation to global gross domestic product (GDP) | **0,67** |
| 1.5.3 Number of countries that adopt and implement national disaster risk reduction strategies in line with the Sendai Framework for Disaster Risk Reduction 2015–2030 | **1,00** |
| 1.5.4 Proportion of local governments that adopt and implement local disaster risk reduction strategies in line with national disaster risk reduction strategies | **1,00** |
| 1.a.1 Total official development assistance grants from all donors that focus on poverty reduction as a share of the recipient country’s gross national income | **1,00** |
| 2.4.1 Proportion of agricultural area under productive and sustainable agriculture | **0,67** |
| 2.a.2 Total official flows (official development assistance plus other official flows) to the agriculture sector | **0,50** |
| 2.c.1 Indicator of food price anomalies | **1,33** |
| 3.3.3 Malaria incidence per 1,000 population | **0,67** |
| 3.6.1 Death rate due to road traffic injuries | **1,33** |
| 3.d.2 Percentage of bloodstream infections due to selected antimicrobial-resistant organisms | **0,67** |
| 4.1.2 Completion rate (primary education, lower secondary education, upper secondary education) | **1,33** |
| 4.2.1 Proportion of children aged 24–59 months who are developmentally on track in health, learning and psychosocial well-being, by sex | **1,00** |
| 4.3.1 Participation rate of youth and adults in formal and non-formal education and training in the previous 12 months, by sex | **1,00** |
| 4.b.1 Volume of official development assistance flows for scholarships by sector and type of study | **1,00** |
| 4.c.1 Proportion of teachers with the minimum required qualifications, by education level | **1,00** |
| 5.3.1 Proportion of women aged 20–24 years who were married or in a union before age 15 and before age 18 | **1,00** |
| 5.5.2 Proportion of women in managerial positions | **1,00** |
| 5.c.1 Proportion of countries with systems to track and make public allocations for gender equality and women’s empowerment | **1,33** |
| 6.3.2 Proportion of bodies of water with good ambient water quality | **1,00** |
| 6.4.1 Change in water-use efficiency over time | **1,00** |
| 6.4.2 Level of water stress: freshwater withdrawal as a proportion of available freshwater resources | **0,67** |
| 6.5.1 Degree of integrated water resources management | **1,00** |
| 6.5.2 Proportion of transboundary basin area with an operational arrangement for water cooperation | **0,67** |
| 6.a.1 Amount of water- and sanitation-related official development assistance that is part of a government-coordinated spending plan | **1,00** |
| 7.2.1 Renewable energy share in the total final energy consumption | **1,33** |
| 7.a.1 International financial flows to developing countries in support of clean energy research and development and renewable energy production, including in hybrid systems | **1,00** |
| 7.b.1 Installed renewable energy-generating capacity in developing countries (in watts per capita) | **1,00** |
| 8.1.1 Annual growth rate of real GDP per capita | **1,33** |
| 8.2.1 Annual growth rate of real GDP per employed person | **1,00** |
| 8.8.2 Level of national compliance with labour rights (freedom of association and collective bargaining) based on International Labour Organization (ILO) textual sources and national legislation, by sex and migrant status | **1,00** |
| 8.b.1 Existence of a developed and operationalized national strategy for youth employment, as a distinct strategy or as part of a national employment strategy | **0,67** |
| 9.4.1 CO2 emission per unit of value added | **0,67** |
| 9.a.1 Total official international support (official development assistance plus other official flows) to infrastructure | **1,33** |
| 10.4.1 Labour share of GDP | **1,00** |
| 10.6.1 Proportion of members and voting rights of developing countries in international organizations | **1,00** |
| 10.7.3 Number of people who died or disappeared in the process of migration towards an international destination | **1,33** |
| 10.b.1 Total resource flows for development, by recipient and donor countries and type of flow (e.g. official development assistance, foreign direct investment and other flows) | **0,50** |
| 11.4.1 Total per capita expenditure on the preservation, protection and conservation of all cultural and natural heritage, by source of funding (public, private), type of heritage (cultural, natural) and level of government (national, regional, and local/municipal) | **1,00** |
| 11.5.2 Direct economic loss in relation to global GDP, damage to critical infrastructure and number of disruptions to basic services, attributed to disasters | **1,50** |
| 11.a.1 Number of countries that have national urban policies or regional development plans that (a) respond to population dynamics; (b) ensure balanced territorial development; and (c) increase local fiscal space | **1,33** |
| 11.b.1 Number of countries that adopt and implement national disaster risk reduction strategies in line with the Sendai Framework for Disaster Risk Reduction 2015–2030 | **0,50** |
| 12.1.1 Number of countries developing, adopting or implementing policy instruments aimed at supporting the shift to sustainable consumption and production | **1,00** |
| 12.4.1 Number of parties to international multilateral environmental agreements on hazardous waste, and other chemicals that meet their commitments and obligations in transmitting information as required by each relevant agreement | **1,00** |
| 12.4.2 (a) Hazardous waste generated per capita; and (b) proportion of hazardous waste treated, by type of treatment | **1,33** |
| 12.5.1 National recycling rate, tons of material recycled | **1,00** |
| 12.8.1 Extent to which (i) global citizenship education and (ii) education for sustainable development are mainstreamed in (a) national education policies; (b) curricula; (c) teacher education; and (d) student assessment | **1,33** |
| 12.a.1 Installed renewable energy-generating capacity in developing countries (in watts per capita) | **1,00** |
| 12.b.1 Implementation of standard accounting tools to monitor the economic and environmental aspects of tourism sustainability | **1,33** |
| 13.1.2 Number of countries that adopt and implement national disaster risk reduction strategies in line with the Sendai Framework for Disaster Risk Reduction 2015–2030 | **1,00** |
| 13.3.1 Extent to which (i) global citizenship education and (ii) education for sustainable development are mainstreamed in (a) national education policies; (b) curricula; (c) teacher education; and (d) student assessment | **1,33** |
| 14.2.1 Number of countries using ecosystem-based approaches to managing marine areas | **1,00** |
| 15.1.2 Proportion of important sites for terrestrial and freshwater biodiversity that are covered by protected areas, by ecosystem type | **1,50** |
| 15.3.1 Proportion of land that is degraded over total land area | **0,50** |
| 15.4.1 Coverage by protected areas of important sites for mountain biodiversity | **1,00** |
| 15.5.1 Red List Index | **1,50** |
| 16.1.1 Number of victims of intentional homicide per 100,000 population, by sex and age | **1,00** |
| 16.1.4 Proportion of population that feel safe walking alone around the area they live | **1,00** |
| 16.5.1 Proportion of persons who had at least one contact with a public official and who paid a bribe to a public official, or were asked for a bribe by those public officials, during the previous 12 months | **1,00** |
| 16.5.2 Proportion of businesses that had at least one contact with a public official and that paid a bribe to a public official, or were asked for a bribe by those public officials during the previous 12 months | **1,00** |
| 16.8.1 Proportion of members and voting rights of developing countries in international organizations | **1,00** |
| 17.1.1 Total government revenue as a proportion of GDP, by source | **1,33** |
| 17.3.2 Volume of remittances (in United States dollars) as a proportion of total GDP | **1,00** |
| 17.6.1 Fixed Internet broadband subscriptions per 100 inhabitants, by speed5 | **1,00** |
| 17.13.1 Macroeconomic Dashboard | **1,00** |
| 17.18.1 Statistical capacity indicator for Sustainable Development Goal monitoring | **1,33** |
