# Supplementary material for: Sustainable Development Goals as a Framework for Teaching and Learning about Health Equity in European Health and Social Care Study Programmes: A Modified Delphi Approach
Source: J Med Syst. 2025 Dec 22;49(1):187. doi: 10.1007/s10916-025-02328-3 (PMC12722463; doi:10.1007/s10916-025-02328-3)
Supplement: Supplementary file 4 — Supplementary Material 4 (DOCX 19.6 KB) [file 10916_2025_2328_MOESM4_ESM.docx]

**SUPPLEMENTARY FILE 4. RESULTS AFTER ROUND 3**

**Supplementary file 4** presents all the indicators included, excluded and re-rated after Round 3.

**Table 4.1.** 50 indicators were included after Round 3.

| *INDICATORS* | *MEAN* |
| --- | --- |
| 1.5.1 Number of deaths, missing persons and directly affected persons attributed to disasters per 100,000 population | **1,25** |
| 1.5.3 Number of countries that adopt and implement national disaster risk reduction strategies in line with the Sendai Framework for Disaster Risk Reduction 2015–2030 | **1,25** |
| 1.a.1 Total official development assistance grants from all donors that focus on poverty reduction as a share of the recipient country’s gross national income | **1** |
| 2.4.1 Proportion of agricultural area under productive and sustainable agriculture | **1,75** |
| 2.c.1 Indicator of food price anomalies | **1,25** |
| 3.3.3 Malaria incidence per 1,000 population | **1** |
| 3.6.1 Death rate due to road traffic injuries | **1,25** |
| 3.d.2 Percentage of bloodstream infections due to selected antimicrobial-resistant organisms | **1,5** |
| 4.1.2 Completion rate (primary education, lower secondary education, upper secondary education) | **2** |
| 4.2.1 Proportion of children aged 24–59 months who are developmentally on track in health, learning and psychosocial well-being, by sex | **1,75** |
| 4.3.1 Participation rate of youth and adults in formal and non-formal education and training in the previous 12 months, by sex | **1,75** |
| 4.b.1 Volume of official development assistance flows for scholarships by sector and type of study | **1,25** |
| 4.c.1 Proportion of teachers with the minimum required qualifications, by education level | **1** |
| 5.3.1 Proportion of women aged 20–24 years who were married or in a union before age 15 and before age 18 | **1,25** |
| 5.5.2 Proportion of women in managerial positions | **1,25** |
| 5.c.1 Proportion of countries with systems to track and make public allocations for gender equality and women’s empowerment | **1,75** |
| 6.3.2 Proportion of bodies of water with good ambient water quality | **1,75** |
| 6.4.2 Level of water stress: freshwater withdrawal as a proportion of available freshwater resources | **1,75** |
| 6.5.1 Degree of integrated water resources management | **1** |
| 6.a.1 Amount of water- and sanitation-related official development assistance that is part of a government-coordinated spending plan | **1,25** |
| 7.2.1 Renewable energy share in the total final energy consumption | **1,5** |
| 7.a.1 International financial flows to developing countries in support of clean energy research and development and renewable energy production, including in hybrid systems | **1,25** |
| 8.1.1 Annual growth rate of real GDP per capita | **1,75** |
| 8.2.1 Annual growth rate of real GDP per employed person | **1,5** |
| 8.8.2 Level of national compliance with labour rights (freedom of association and collective bargaining) based on International Labour Organization (ILO) textual sources and national legislation, by sex and migrant status | **1,75** |
| 8.b.1 Existence of a developed and operationalized national strategy for youth employment, as a distinct strategy or as part of a national employment strategy | **1,75** |
| 9.4.1 CO2 emission per unit of value added | **1,5** |
| 9.a.1 Total official international support (official development assistance plus other official flows) to infrastructure | **1,25** |
| 10.4.1 Labour share of GDP | **1** |
| 10.6.1 Proportion of members and voting rights of developing countries in international organizations | **1** |
| 10.7.3 Number of people who died or disappeared in the process of migration towards an international destination | **1,5** |
| 10.b.1 Total resource flows for development, by recipient and donor countries and type of flow (e.g. official development assistance, foreign direct investment and other flows) | **1** |
| 11.5.2 Direct economic loss in relation to global GDP, damage to critical infrastructure and number of disruptions to basic services, attributed to disasters | **1,25** |
| 11.a.1 Number of countries that have national urban policies or regional development plans that (a) respond to population dynamics; (b) ensure balanced territorial development; and (c) increase local fiscal space | **1,75** |
| 12.1.1 Number of countries developing, adopting or implementing policy instruments aimed at supporting the shift to sustainable consumption and production | **1,5** |
| 12.4.1 Number of parties to international multilateral environmental agreements on hazardous waste, and other chemicals that meet their commitments and obligations in transmitting information as required by each relevant agreement | **1,25** |
| 12.4.2 (a) Hazardous waste generated per capita; and (b) proportion of hazardous waste treated, by type of treatment | **1,75** |
| 12.5.1 National recycling rate, tons of material recycled | **1,75** |
| 12.8.1 Extent to which (i) global citizenship education and (ii) education for sustainable development are mainstreamed in (a) national education policies; (b) curricula; (c) teacher education; and (d) student assessment | **2** |
| 12.a.1 Installed renewable energy-generating capacity in developing countries (in watts per capita) | **1,25** |
| 13.3.1 Extent to which (i) global citizenship education and (ii) education for sustainable development are mainstreamed in (a) national education policies; (b) curricula; (c) teacher education; and (d) student assessment | **2** |
| 14.2.1 Number of countries using ecosystem-based approaches to managing marine areas | **1,25** |
| 15.1.2 Proportion of important sites for terrestrial and freshwater biodiversity that are covered by protected areas, by ecosystem type | **2** |
| 15.3.1 Proportion of land that is degraded over total land area | **1,25** |
| 15.4.1 Coverage by protected areas of important sites for mountain biodiversity | **1,25** |
| 16.1.1 Number of victims of intentional homicide per 100,000 population, by sex and age | **1,25** |
| 16.1.4 Proportion of population that feel safe walking alone around the area they live | **1,25** |
| 17.1.1 Total government revenue as a proportion of GDP, by source | **1,5** |
| 17.6.1 Fixed Internet broadband subscriptions per 100 inhabitants, by speed5 | **1,75** |
| 17.18.1 Statistical capacity indicator for Sustainable Development Goal monitoring | **1,25** |

**Table 4.2.** 16 indicators were excluded after Round 3.

| *INDICATORS* | *MEAN* |
| --- | --- |
| 1.5.2 Direct economic loss attributed to disasters in relation to global gross domestic product (GDP) | **0,75** |
| 1.5.4 Proportion of local governments that adopt and implement local disaster risk reduction strategies in line with national disaster risk reduction strategies | **0,75** |
| 2.a.2 Total official flows (official development assistance plus other official flows) to the agriculture sector | **0,5** |
| 6.4.1 Change in water-use efficiency over time | **0,5** |
| 6.5.2 Proportion of transboundary basin area with an operational arrangement for water cooperation | **0,25** |
| 7.b.1 Installed renewable energy-generating capacity in developing countries (in watts per capita) | **0,75** |
| 11.4.1 Total per capita expenditure on the preservation, protection and conservation of all cultural and natural heritage, by source of funding (public, private), type of heritage (cultural, natural) and level of government (national, regional, and local/municipal) | **0,5** |
| 11.b.1 Number of countries that adopt and implement national disaster risk reduction strategies in line with the Sendai Framework for Disaster Risk Reduction 2015–2030 | **0,75** |
| 12.b.1 Implementation of standard accounting tools to monitor the economic and environmental aspects of tourism sustainability | **0,75** |
| 13.1.2 Number of countries that adopt and implement national disaster risk reduction strategies in line with the Sendai Framework for Disaster Risk Reduction 2015–2030 | **0,75** |
| 15.5.1 Red List Index | **0,5** |
| 16.5.1 Proportion of persons who had at least one contact with a public official and who paid a bribe to a public official, or were asked for a bribe by those public officials, during the previous 12 months | **0,5** |
| 16.5.2 Proportion of businesses that had at least one contact with a public official and that paid a bribe to a public official, or were asked for a bribe by those public officials during the previous 12 months | **0,5** |
| 16.8.1 Proportion of members and voting rights of developing countries in international organizations | **0,25** |
| 17.3.2 Volume of remittances (in United States dollars) as a proportion of total GDP | **0,75** |
| 17.13.1 Macroeconomic Dashboard | **0,5** |
